# Supplementary material for: Developing mitochondrial base editors with diverse context compatibility and high fidelity via saturated spacer library
Source: Nat Commun. 2023 Oct 19;14:6625. doi: 10.1038/s41467-023-42359-3 (PMC10587121; doi:10.1038/s41467-023-42359-3)
Supplement: Supplementary file 1 — Supplementary Information [file 41467_2023_42359_MOESM1_ESM.pdf]

Supplementary information

Developing mitochondrial base editors with diverse context compatibility and high fidelity via saturated spacer library

Haifeng Sun[1#], Zhaojun Wang[1#], Limini Shen[1#], Yeling Feng[1#], Lu Han[1], Xuezhen Qian[1], Runde Meng[1], Kangming Ji[1], Dong Liang[2], Fei Zhou[3], Xin Lou[4*], Jun Zhang[1*], Bin Shen[1*]

[1]State Key Laboratory of Reproductive Medicine and Offspring Health, Women's Hospital of Nanjing Medical University, Nanjing Maternity and Child Health Care Hospital, Center for Global Health, Gusu School, Nanjing Medical University, Nanjing 211166, China.
[2]Department of Prenatal Diagnosis, Women's Hospital of Nanjing Medical University, Nanjing Maternity and Child Health Care Hospital, Nanjing, Jiangsu, China.
[3]Cambridge-Suda Genomic Resource Center, Suzhou Medical College of Soochow University, Suzhou 215123, China
[4]Research Institute of Intelligent Computing, Zhejiang Lab, Hangzhou 311100, China

**Corresponding authors:*
Bin Shen, E-mail: binshen@njmu.edu.cn
Jun Zhang, E-mail: zhang_jun@njmu.edu.cn
Xin Lou, E-mail: xin.lou@zhejianglab.edu.cn

[#]These authors contribute equally to this work.

Supplementary information

Contents

Supplementary Fig. 1 | All sequence contexts and coverage of the saturated spacer library.

Supplementary Fig. 2 | Editing properties of DddA homologs in the spacer library.

Supplementary Fig. 3 | Off-target editing induced by TALE-free DddA homologs on mtDNA.

Supplementary Fig. 4 | FZY2-DdCBEs mediated GC editing at m.G3635 and m.G8313.

Supplementary Fig. 5 | Engineering FZY2 to enhance its GC deaminase activity.

Supplementary Fig. 6 | Homologous protein retrieval of DddIAS.

Supplementary Fig. 7 | New mtDNA base editors with diversiform NC preference.

Supplementary Fig. 8 | Gating strategy for EGFP/mCherry double positive cells.

Supplementary Table 1. Spacer Library.

Supplementary Table 2. Summary of reported C/G to T/A conversions on human mtDNA.

Supplementary Table 3. Plasmid suite containing m.A8344G mutation.

Supplementary Table 4. Primers.

Supplementary Table 5. C/G to T/A SNPs on HEK293FT mtDNA.

Supplementary Note. Amino acid sequences used in this study.

Supplementary Fig. 1

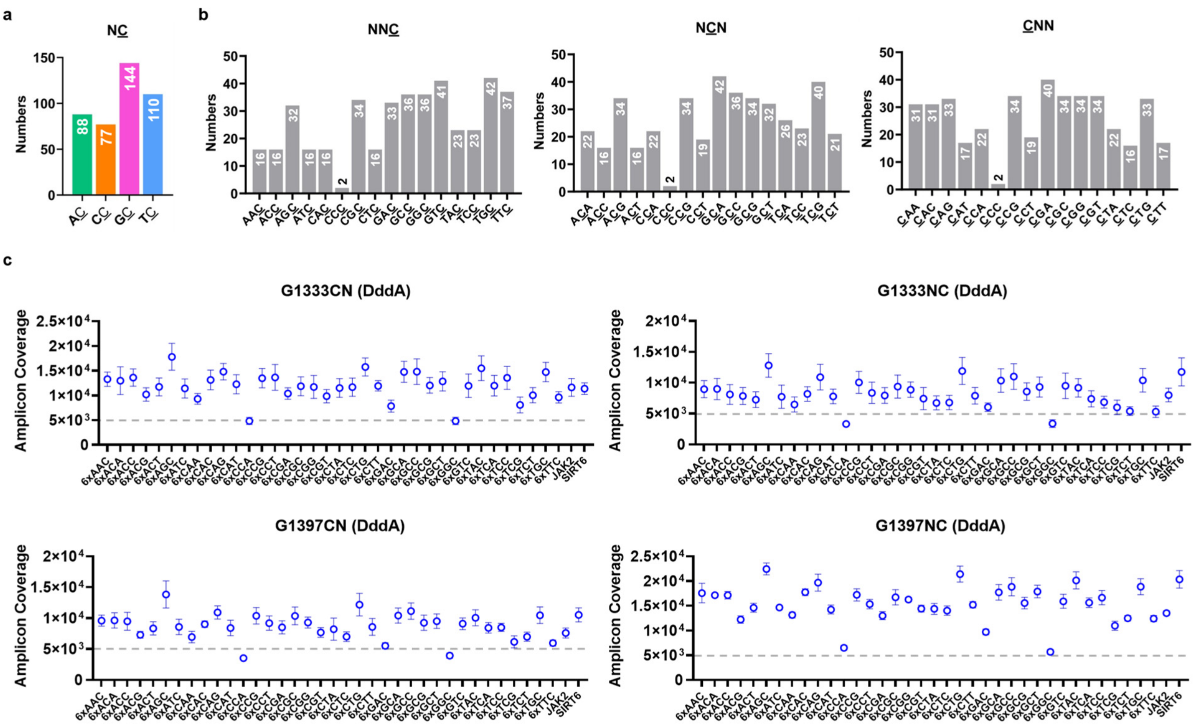

Supplementary Fig. 1 | All sequence contexts and coverage of the saturated spacer library. **a**, The number of NCs in the saturated spacer library. **b**, The number of NNCs, NCNs, and CNNs in the saturated spacer library. **c**, The coverage of each spacer in one reaction of amplicon sequencing. Taking DddA-DdCBEs (G1333CN, G1333NC, G1397CN and G1397NC) as examples, the coverage of vast majority of spacer is great than 5,000x (grey dashed line). Values and error bars reflect the mean ± SD of n = 3 independent biological replicates. Source data are provided as a Source Data file.

Supplementary Fig. 2

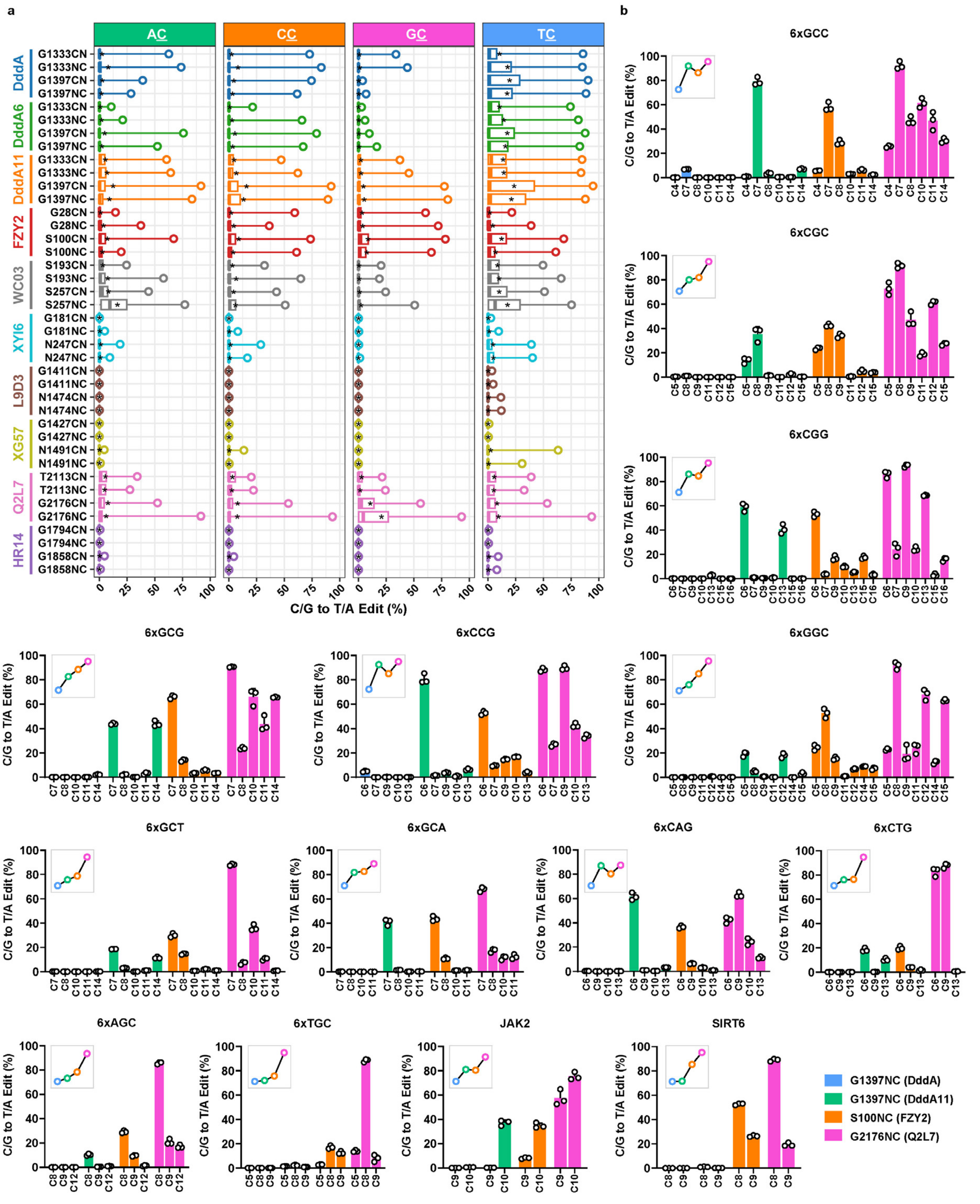

Supplementary Fig. 2 | Editing properties of DddA homologs in the spacer library.

a, The maximum, minimum, upper quartile, lower quartile, and median C/G to T/A editing

efficiencies (%) in the spacer library mediated by DddA and its homologs. Asterisk represents the average editing efficiency. **b**, The edited GC̲s beyond 10% (C/G to T/A editing) induced by DddA-, DddA11-, FZY2- and Q2L7-DdCBEs are selected for visualization. The insets indicate the max editing efficiency (%) of each DdCBE within the spacing region. Values and error bars reflect the mean ± SD of *n* = 3 independent biological replicates. Source data are provided as a Source Data file.

Supplementary Fig. 3

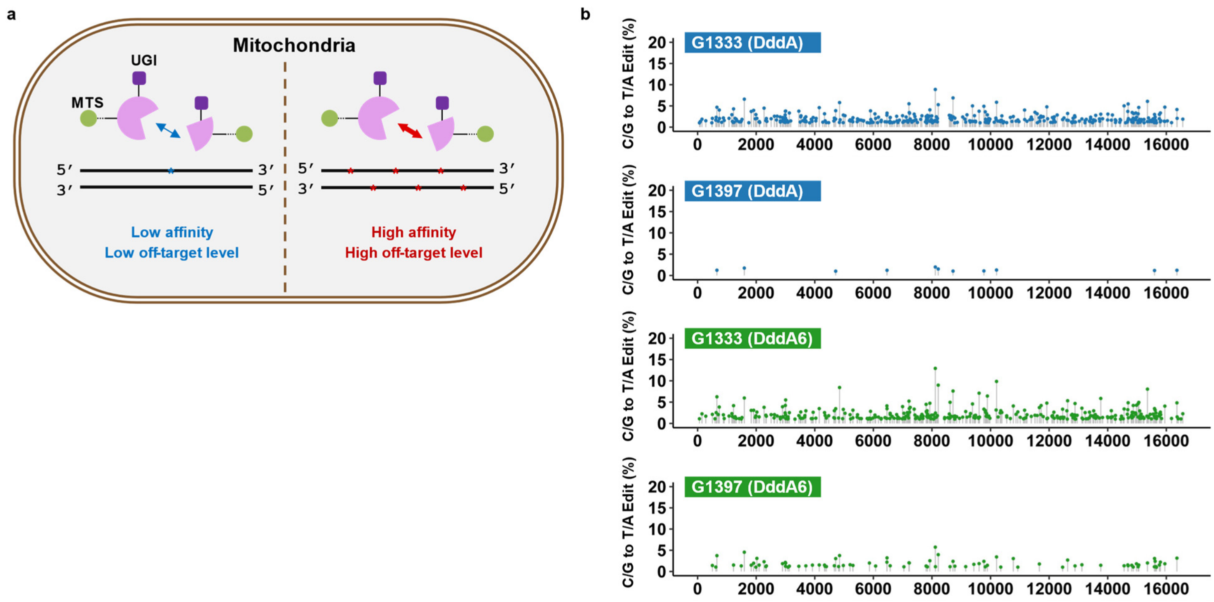

Supplementary Fig. 3 | Off-target editing induced by TALE-free DddA homologs on mtDNA. **a**, Spontaneous assembly of TALE-free $DddA_{tox}$ halves can induce off-target editing on mtDNA. A higher affinity of the two halves would result in more off-target editing. **b**, Whole mtDNA off-target plots of TALE-free DddA and DddA6. Values reflect the mean of *n* = 2 independent biological replicates. Source data are provided as a Source Data file.

Supplementary Fig. 4

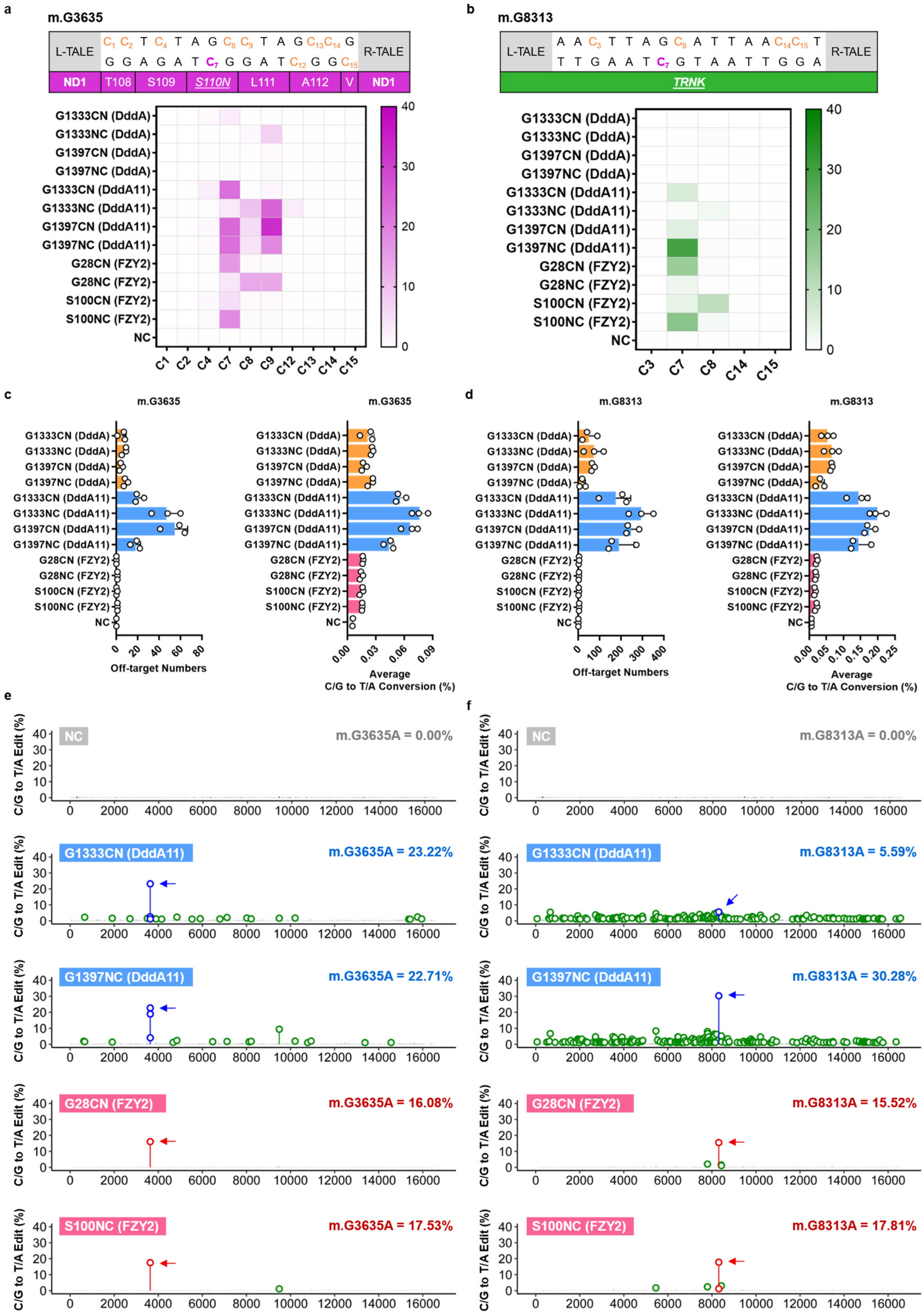

Supplementary Fig. 4 | FZY2-DdCBEs mediated GC editing at m.G3635 and m.G8313.

a, Installation of m.G3635A (C_7) using DddA-, DddA11- and FZY2-DdCBEs. **b**, Installation of m.G8313A (C_7) using DddA-, DddA11- and FZY2-DdCBEs.**c** and **d**, Off-target numbers and average C/G to T/A conversion rate (%) on mtDNA induced by DddA-, DddA11- and FZY2-DdCBEs targeting m.G3635 (**c**) and m.G8313 (**d**). **e** and **f**, Off-target sites on mtDNA induced by DddA11- and FZY2-DdCBEs targeting m.G3635 (**e**) and m.G8313 (**f**). Off-target sites are in green. Edited Cs within spacing region induced by DddA11-DdCBEs are in blue, edited Cs induced by FZY2-DdCBEs are in red. The target sites are marked by arrows. The editing efficiency of the target site is marked in the upper right corner. Untreated cells are used as negative control (NC). **a**, **b**, **e** and **f**, Values reflect the mean of $n = 3$ independent biological replicates. **c** and **d**, Values and error bars reflect the mean ± SD of $n = 3$ independent biological replicates. $n = 2$ independent biological replicates for NC group in **a** and **c**. Source data are provided as a Source Data file.

Supplementary Fig. 5

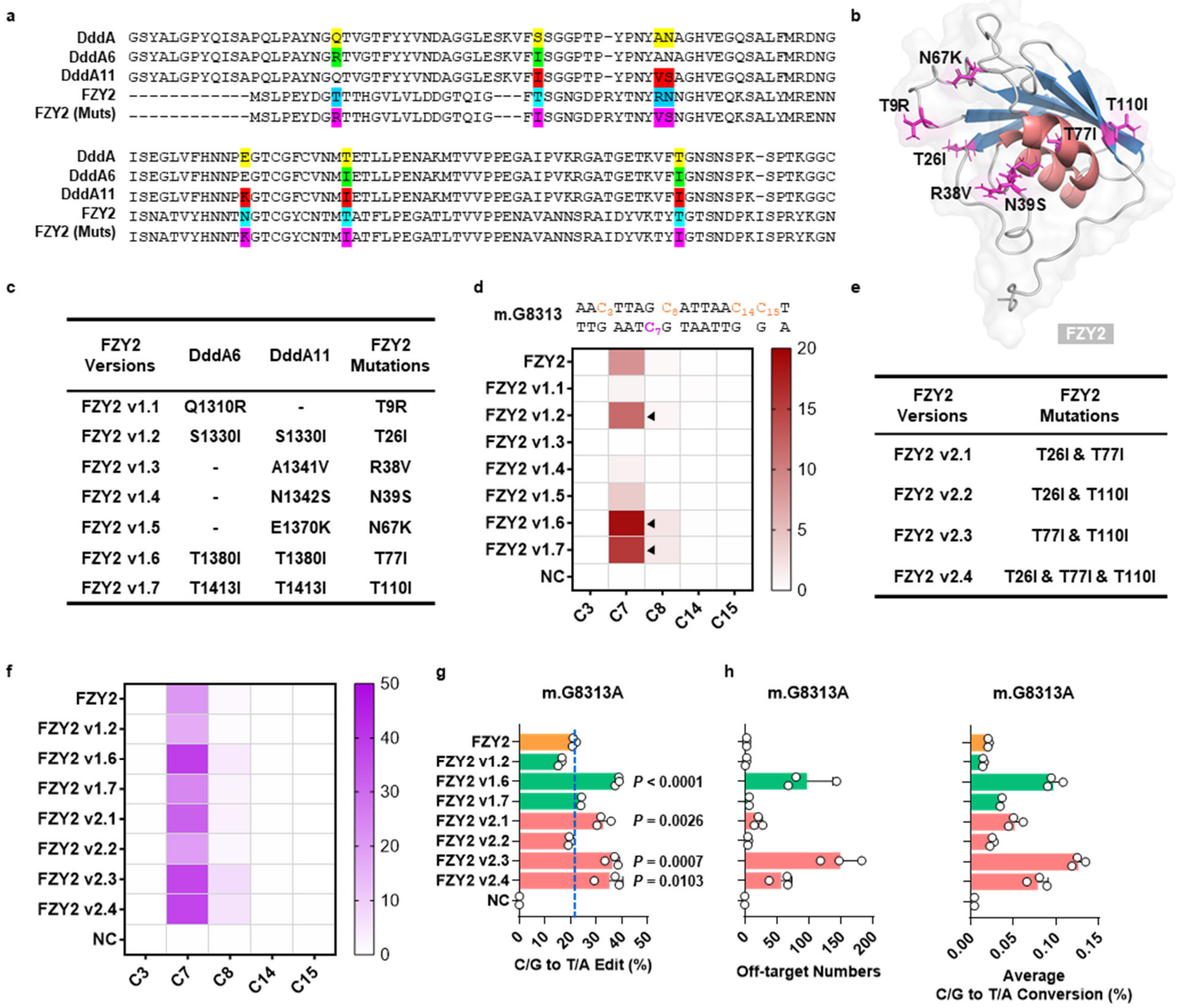

FZY2 Versions	DddA6	DddA11	FZY2 Mutations
FZY2 v1.1	Q1310R	-	T9R
FZY2 v1.2	S1330I	S1330I	T26I
FZY2 v1.3	-	A1341V	R38V
FZY2 v1.4	-	N1342S	N39S
FZY2 v1.5	-	E1370K	N67K
FZY2 v1.6	T1380I	T1380I	T77I
FZY2 v1.7	T1413I	T1413I	T110I

FZY2 Versions	FZY2 Mutations
FZY2 v2.1	T26I & T77I
FZY2 v2.2	T26I & T110I
FZY2 v2.3	T77I & T110I
FZY2 v2.4	T26I & T77I & T110I

Supplementary Fig. 5 | Engineering FZY2 to enhance its GC deaminase activity.

a, Multiple sequence alignment of DddA, DddA6, DddA11 and FZY2. The engineered amino acids are marked in green, red and purple for DddA6, DddA11 and mutant FZY2, respectively, and the corresponding original amino acids are marked in yellow and blue for DddA and FZY2. **b**, Substituted amino acids in FZY2 are indicated by purple sticks in 3D structure. α-helixes are colored in rose-bengal and β-sheets in blue. **c**, Single amino acid substitution for FZY2 in the first round of engineering. **d**, Editing efficiencies (%) of FZY2-DdCBE and its variants at m.G8313 (C_7). Three FZY2-DdCBE variants with higher activities (marked by arrowhead) were selected for the next round of engineering. **e**, Double and triple amino acid substitutions for FZY2 in the second round of engineering. **f**, Editing efficiencies (%) of FZY2-DdCBE and its variants at m.G8313. **g**,

Quantification of editing efficiencies of FZY2 and its variants at target site. *P* values are calculated by comparing with FZY2-DdCBE.**h**, Off-target numbers and average C/G to T/A conversion rate (%) on mtDNA induced by FZY2-DdCBE and its variants at m.G8313.

d and **f**, Values reflect the mean of n = 3 independent biological replicates. **g** and **h**, Values and error bars reflect the mean ± SD of n = 3 independent biological replicates. n = 2 independent biological replicates for FZY2 v1.7 and NC groups in **f-h**. *P* values were calculated by Student's unpaired two-tailed *t*-test. Source data are provided as a Source Data file.

Supplementary Fig. 6

a

ID	DddA Name	DddA Tax	DddI$_A$ Name	DddI$_A$ Tax	DddI$_A$ Rename
0	DddA	Burkholderia cenocepacia (strain H111)	P0DUH6	Burkholderia cenocepacia (strain H111)	DddA-DddI$_A$
1	FZY2	Lachnospiraceae bacterium sunii NSJ-8	A0A7G9FY10	Lachnospiraceae bacterium sunii NSJ-8	FZY2-DddI$_A$
2	WC03	Ruminococcus sp. AF17-6	A0A373WEI4	Ruminococcus sp. AF17-6	WC03-DddI$_A$
3	L9D3	Paraburkholderia guartelaensis	A0A4V2ZVD0	Paraburkholderia guartelaensis	L9D3-DddI$_A$
4	XG57	Burkholderia ubonensis	A0A105KEA0	Burkholderia ubonensis	XG57-DddI$_A$
5	HR14	Propionibacterium acidifaciens	A0A7X6NS71	Propionibacterium sp.	HR14-DddI$_A$

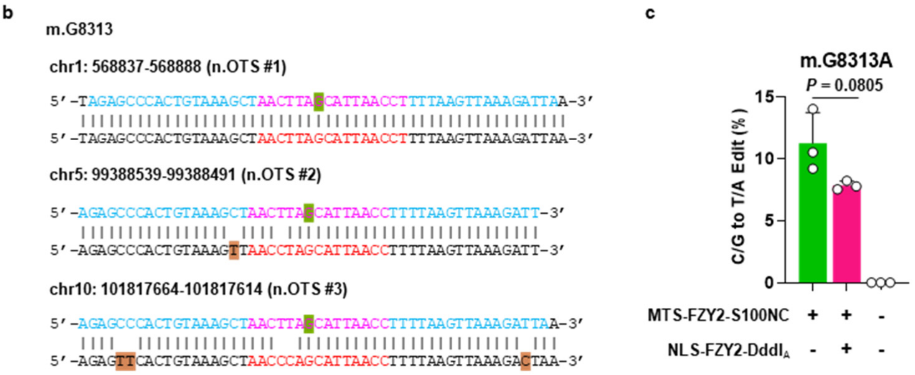

Supplementary Fig. 6 | Homologous protein retrieval of DddI$_A$s.

a, Information of five DddI$_A$s. **b**, The predicted n.OTSs when targeting m.G8313. Target sites are in green, mismatches in TALE recognition regions in orange. **c**, Editing efficiencies (%) at m.G8313A induced by FZY2-DdCBE with or without FZY2-DddI$_A$. Values and error bars reflect the mean ± SD of $n = 3$ independent biological replicates. P values were calculated by Student's unpaired two-tailed t-test. Source data are provided as a Source Data file.

Supplementary Fig. 7

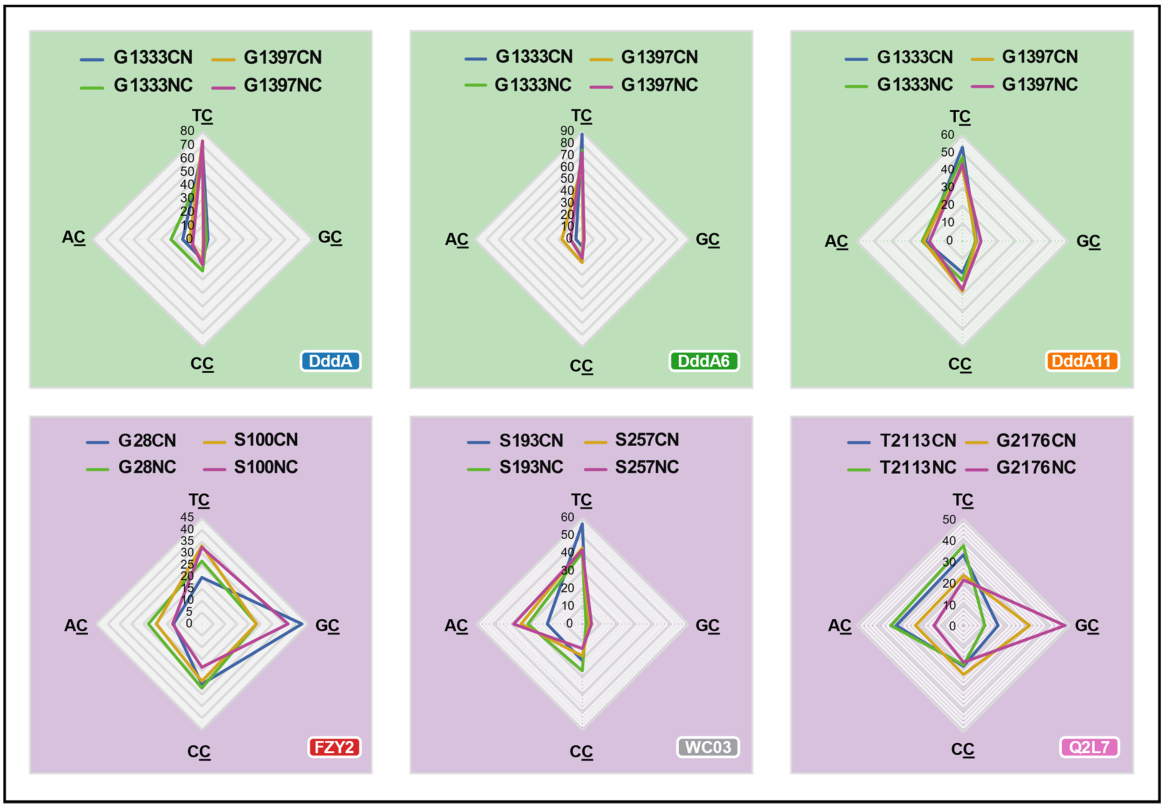

Supplementary Fig. 7 | New mtDNA base editors with diversiform NC preference.

Radar maps indicate the NC preference of DdCBEs derived from newly identified DddA homologs in this study. Values reflect the mean of n = 3 independent biological replicates. Source data are provided as a Source Data file.

Supplementary Fig. 8

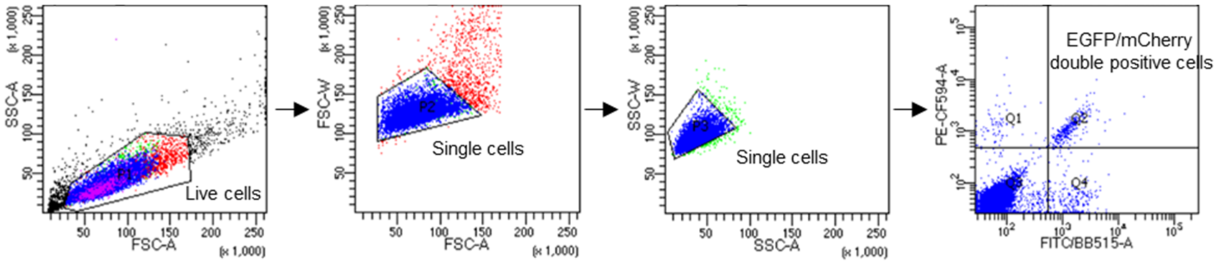

Supplementary Fig. 8 | Gating strategy for EGFP/mCherry double positive cells (cf. Figure 7e).

Supplementary Table 1. Spacer Library.

<table>
<tr><th>ID</th><th>Spacer Name</th><th>Barcode</th><th>Spacer Sequence</th><th>Spacer Length</th><th>Amplicon Length</th></tr>
<tr><td>1</td><td>6xAAC</td><td>GCTT</td><td>AACAACAACAACAACAAC</td><td>18</td><td>384</td></tr>
<tr><td colspan="6">NNNGATCACTCTCGGCATGGACGAGCTGTACAAGTAAAGCGCTTGGCCGCGACTCTAGAT
CATAATCAGCCACCAGACTTAACAACAACAACAACAACACAGCTGGTACCACATTTGTAG
AGGTTTTACTTGCTTTAAAAAACCTCCCACACCTCCCCCTGAACCTGAAACATAAAGCGAC
TCTAGATCATAATCAGCCATACCACATTTGTAGAGGTTTTACTTGCTTTAAAAAACCTCCC
ACACCTCCCCCTGAACCTGAAACATAAAATGAATGCAATTGTTGTTGTTAACTTGTTTATT
GCAGCTTATAATGGTTACAAATAAAGCAATAGCATCACAAATTTCACAAATAAAGCATTT
TTTTCACTGCATTCTAGTTGTGGTNNN</td></tr>
<tr><td>2</td><td>6xACA</td><td>GTTA</td><td>ACAACAACAACAACAACA</td><td>18</td><td>384</td></tr>
<tr><td colspan="6">NNNGATCACTCTCGGCATGGACGAGCTGTACAAGTAAAGCGTTAGGCCGCGACTCTAGAT
CATAATCAGCCACCAGACTTACAACAACAACAACAACAACAGCTGGTACCACATTTGTAG
AGGTTTTACTTGCTTTAAAAAACCTCCCACACCTCCCCCTGAACCTGAAACATAAAGCGAC
TCTAGATCATAATCAGCCATACCACATTTGTAGAGGTTTTACTTGCTTTAAAAAACCTCCC
ACACCTCCCCCTGAACCTGAAACATAAAATGAATGCAATTGTTGTTGTTAACTTGTTTATT
GCAGCTTATAATGGTTACAAATAAAGCAATAGCATCACAAATTTCACAAATAAAGCATTT
TTTTCACTGCATTCTAGTTGTGGTNNN</td></tr>
<tr><td>3</td><td>6xACC</td><td>TATA</td><td>ACCACCACCACCACCACC</td><td>18</td><td>384</td></tr>
<tr><td colspan="6">NNNGATCACTCTCGGCATGGACGAGCTGTACAAGTAAAGCTATAGGCCGCGACTCTAGAT
CATAATCAGCCACCAGACTTACCACCACCACCACCACCACAGCTGGTACCACATTTGTAG
AGGTTTTACTTGCTTTAAAAAACCTCCCACACCTCCCCCTGAACCTGAAACATAAAGCGAC
TCTAGATCATAATCAGCCATACCACATTTGTAGAGGTTTTACTTGCTTTAAAAAACCTCCC
ACACCTCCCCCTGAACCTGAAACATAAAATGAATGCAATTGTTGTTGTTAACTTGTTTATT
GCAGCTTATAATGGTTACAAATAAAGCAATAGCATCACAAATTTCACAAATAAAGCATTT
TTTTCACTGCATTCTAGTTGTGGTNNN</td></tr>
<tr><td>4</td><td>6xACG</td><td>TGCC</td><td>ACGACGACGACGACGACG</td><td>18</td><td>384</td></tr>
<tr><td colspan="6">NNNGATCACTCTCGGCATGGACGAGCTGTACAAGTAAAGCTGCCGGCCGCGACTCTAGAT
CATAATCAGCCACCAGACTTACGACGACGACGACGACGACAGCTGGTACCACATTTGTAG
AGGTTTTACTTGCTTTAAAAAACCTCCCACACCTCCCCCTGAACCTGAAACATAAAGCGAC
TCTAGATCATAATCAGCCATACCACATTTGTAGAGGTTTTACTTGCTTTAAAAAACCTCCC
ACACCTCCCCCTGAACCTGAAACATAAAATGAATGCAATTGTTGTTGTTAACTTGTTTATT
GCAGCTTATAATGGTTACAAATAAAGCAATAGCATCACAAATTTCACAAATAAAGCATTT
TTTTCACTGCATTCTAGTTGTGGTNNN</td></tr>
<tr><td>5</td><td>6xACT</td><td>AACC</td><td>ACTACTACTACTACTACT</td><td>18</td><td>384</td></tr>
<tr><td colspan="6">NNNGATCACTCTCGGCATGGACGAGCTGTACAAGTAAAGCAACCGGCCGCGACTCTAGAT
CATAATCAGCCACCAGACTTACTACTACTACTACTACTACAGCTGGTACCACATTTGTAGA
GGTTTTACTTGCTTTAAAAAACCTCCCACACCTCCCCCTGAACCTGAAACATAAAGCGACT
CTAGATCATAATCAGCCATACCACATTTGTAGAGGTTTTACTTGCTTTAAAAAACCTCCCA
CACCTCCCCCTGAACCTGAAACATAAAATGAATGCAATTGTTGTTGTTAACTTGTTTATTG
CAGCTTATAATGGTTACAAATAAAGCAATAGCATCACAAATTTCACAAATAAAGCATTTTT
TTCACTGCATTCTAGTTGTGGTNNN</td></tr>
<tr><td>6</td><td>6xAGC</td><td>TTGG</td><td>AGCAGCAGCAGCAGCAGC</td><td>18</td><td>384</td></tr>
</table>

NNNGATCACTCTCGGCATGGACGAGCTGTACAAGTAAAGCTTGGGGCCGCGACTCTAGATCATAATCAGCCACCAGACTTAGCAGCAGCAGCAGCACAGCTGGTACCACATTTGTAGAGGTTTTACTTGCTTTAAAAAACCTCCCACACCTCCCCCTGAACCTGAAACATAAAGCGACTCTAGATCATAATCAGCCATACCACATTTGTAGAGGTTTTACTTGCTTTAAAAAACCTCCCACACCTCCCCCTGAACCTGAAACATAAAATGAATGCAATTGTTGTTGTTAACTTGTTTATTGCAGCTTATAATGGTTACAAATAAAGCAATAGCATCACAAATTTCACAAATAAAGCATTTTTTTCACTGCATTCTAGTTGTGGTNNN					
7	6xATC	CCAA	ATCATCATCATCATCATC	18	384
NNNGATCACTCTCGGCATGGACGAGCTGTACAAGTAAAGCCCAAGGCCGCGACTCTAGATCATAATCAGCCACCAGACTTATCATCATCATCATCATCACAGCTGGTACCACATTTGTAGAGGTTTTACTTGCTTTAAAAAACCTCCCACACCTCCCCCTGAACCTGAAACATAAAGCGACTCTAGATCATAATCAGCCATACCACATTTGTAGAGGTTTTACTTGCTTTAAAAAACCTCCCACACCTCCCCCTGAACCTGAAACATAAAATGAATGCAATTGTTGTTGTTAACTTGTTTATTGCAGCTTATAATGGTTACAAATAAAGCAATAGCATCACAAATTTCACAAATAAAGCATTTTTTTCACTGCATTCTAGTTGTGGTNNN					
8	6xCAA	AGTT	CAACAACAACAACAACAA	18	384
NNNGATCACTCTCGGCATGGACGAGCTGTACAAGTAAAGCAGTTGGCCGCGACTCTAGATCATAATCAGCCACCAGACTTCAACAACAACAACAACAAACAGCTGGTACCACATTTGTAGAGGTTTTACTTGCTTTAAAAAACCTCCCACACCTCCCCCTGAACCTGAAACATAAAGCGACTCTAGATCATAATCAGCCATACCACATTTGTAGAGGTTTTACTTGCTTTAAAAAACCTCCCACACCTCCCCCTGAACCTGAAACATAAAATGAATGCAATTGTTGTTGTTAACTTGTTTATTGCAGCTTATAATGGTTACAAATAAAGCAATAGCATCACAAATTTCACAAATAAAGCATTTTTTTCACTGCATTCTAGTTGTGGTNNN					
9	6xCAC	ACCT	CACCACCACCACCACCAC	18	384
NNNGATCACTCTCGGCATGGACGAGCTGTACAAGTAAAGCACCTGGCCGCGACTCTAGATCATAATCAGCCACCAGACTTCACCACCACCACCACCACACAGCTGGTACCACATTTGTAGAGGTTTTACTTGCTTTAAAAAACCTCCCACACCTCCCCCTGAACCTGAAACATAAAGCGACTCTAGATCATAATCAGCCATACCACATTTGTAGAGGTTTTACTTGCTTTAAAAAACCTCCCACACCTCCCCCTGAACCTGAAACATAAAATGAATGCAATTGTTGTTGTTAACTTGTTTATTGCAGCTTATAATGGTTACAAATAAAGCAATAGCATCACAAATTTCACAAATAAAGCATTTTTTTCACTGCATTCTAGTTGTGGTNNN					
10	6xCAG	CTTA	CAGCAGCAGCAGCAGCAG	18	384
NNNGATCACTCTCGGCATGGACGAGCTGTACAAGTAAAGCCTTAGGCCGCGACTCTAGATCATAATCAGCCACCAGACTTCAGCAGCAGCAGCAGCAGACAGCTGGTACCACATTTGTAGAGGTTTTACTTGCTTTAAAAAACCTCCCACACCTCCCCCTGAACCTGAAACATAAAGCGACTCTAGATCATAATCAGCCATACCACATTTGTAGAGGTTTTACTTGCTTTAAAAAACCTCCCACACCTCCCCCTGAACCTGAAACATAAAATGAATGCAATTGTTGTTGTTAACTTGTTTATTGCAGCTTATAATGGTTACAAATAAAGCAATAGCATCACAAATTTCACAAATAAAGCATTTTTTTCACTGCATTCTAGTTGTGGTNNN					
11	6xCAT	CAGT	CATCATCATCATCATCAT	18	384
NNNGATCACTCTCGGCATGGACGAGCTGTACAAGTAAAGCCAGTGGCCGCGACTCTAGATCATAATCAGCCACCAGACTTCATCATCATCATCATCATACAGCTGGTACCACATTTGTAGAGGTTTTACTTGCTTTAAAAAACCTCCCACACCTCCCCCTGAACCTGAAACATAAAGCGACTCTAGATCATAATCAGCCATACCACATTTGTAGAGGTTTTACTTGCTTTAAAAAACCTCCCACACCTCCCCCTGAACCTGAAACATAAAATGAATGCAATTGTTGTTGTTAACTTGTTTATTG					

<table>
<tr><td colspan="6">CAGCTTATAATGGTTACAAATAAAGCAATAGCATCACAAATTTCACAAATAAAGCATTTTT
TTCACTGCATTCTAGTTGTGGTNNN</td></tr>
<tr><td>12</td><td>6xCCA</td><td>ATAT</td><td>CCACCACCACCACCACCA</td><td>18</td><td>384</td></tr>
<tr><td colspan="6">NNNGATCACTCTCGGCATGGACGAGCTGTACAAGTAAAGCATATGGCCGCGACTCTAGAT
CATAATCAGCCACCAGACTTCCACCACCACCACCACCAACAGCTGGTACCACATTTGTAG
AGGTTTTACTTGCTTTAAAAAACCTCCCACACCTCCCCCTGAACCTGAAACATAAAGCGAC
TCTAGATCATAATCAGCCATACCACATTTGTAGAGGTTTTACTTGCTTTAAAAAACCTCCC
ACACCTCCCCCTGAACCTGAAACATAAAATGAATGCAATTGTTGTTGTTAACTTGTTTATT
GCAGCTTATAATGGTTACAAATAAAGCAATAGCATCACAAATTTCACAAATAAAGCATTT
TTTTCACTGCATTCTAGTTGTGGTNNN</td></tr>
<tr><td>13</td><td>6xCCG</td><td>ATAG</td><td>CCGCCGCCGCCGCCGCCG</td><td>18</td><td>384</td></tr>
<tr><td colspan="6">NNNGATCACTCTCGGCATGGACGAGCTGTACAAGTAAAGCATAGGGCCGCGACTCTAGAT
CATAATCAGCCACCAGACTTCCGCCGCCGCCGCCGCCGACAGCTGGTACCACATTTGTAG
AGGTTTTACTTGCTTTAAAAAACCTCCCACACCTCCCCCTGAACCTGAAACATAAAGCGAC
TCTAGATCATAATCAGCCATACCACATTTGTAGAGGTTTTACTTGCTTTAAAAAACCTCCC
ACACCTCCCCCTGAACCTGAAACATAAAATGAATGCAATTGTTGTTGTTAACTTGTTTATT
GCAGCTTATAATGGTTACAAATAAAGCAATAGCATCACAAATTTCACAAATAAAGCATTT
TTTTCACTGCATTCTAGTTGTGGTNNN</td></tr>
<tr><td>14</td><td>6xCCT</td><td>CCTG</td><td>CCTCCTCCTCCTCCTCCT</td><td>18</td><td>384</td></tr>
<tr><td colspan="6">NNNGATCACTCTCGGCATGGACGAGCTGTACAAGTAAAGCCCTGGGCCGCGACTCTAGAT
CATAATCAGCCACCAGACTTCCTCCTCCTCCTCCTCCTACAGCTGGTACCACATTTGTAGA
GGTTTTACTTGCTTTAAAAAACCTCCCACACCTCCCCCTGAACCTGAAACATAAAGCGACT
CTAGATCATAATCAGCCATACCACATTTGTAGAGGTTTTACTTGCTTTAAAAAACCTCCCA
CACCTCCCCCTGAACCTGAAACATAAAATGAATGCAATTGTTGTTGTTAACTTGTTTATTG
CAGCTTATAATGGTTACAAATAAAGCAATAGCATCACAAATTTCACAAATAAAGCATTTTT
TTCACTGCATTCTAGTTGTGGTNNN</td></tr>
<tr><td>15</td><td>6xCGA</td><td>ACTG</td><td>CGACGACGACGACGACGA</td><td>18</td><td>384</td></tr>
<tr><td colspan="6">NNNGATCACTCTCGGCATGGACGAGCTGTACAAGTAAAGCACTGGGCCGCGACTCTAGAT
CATAATCAGCCACCAGACTTCGACGACGACGACGACGAACAGCTGGTACCACATTTGTAG
AGGTTTTACTTGCTTTAAAAAACCTCCCACACCTCCCCCTGAACCTGAAACATAAAGCGAC
TCTAGATCATAATCAGCCATACCACATTTGTAGAGGTTTTACTTGCTTTAAAAAACCTCCC
ACACCTCCCCCTGAACCTGAAACATAAAATGAATGCAATTGTTGTTGTTAACTTGTTTATT
GCAGCTTATAATGGTTACAAATAAAGCAATAGCATCACAAATTTCACAAATAAAGCATTT
TTTTCACTGCATTCTAGTTGTGGTNNN</td></tr>
<tr><td>16</td><td>6xCGC</td><td>ACAC</td><td>CGCCGCCGCCGCCGCCGC</td><td>18</td><td>384</td></tr>
<tr><td colspan="6">NNNGATCACTCTCGGCATGGACGAGCTGTACAAGTAAAGCACACGGCCGCGACTCTAGAT
CATAATCAGCCACCAGACTTCGCCGCCGCCGCCGCCGCACAGCTGGTACCACATTTGTAG
AGGTTTTACTTGCTTTAAAAAACCTCCCACACCTCCCCCTGAACCTGAAACATAAAGCGAC
TCTAGATCATAATCAGCCATACCACATTTGTAGAGGTTTTACTTGCTTTAAAAAACCTCCC
ACACCTCCCCCTGAACCTGAAACATAAAATGAATGCAATTGTTGTTGTTAACTTGTTTATT
GCAGCTTATAATGGTTACAAATAAAGCAATAGCATCACAAATTTCACAAATAAAGCATTT
TTTTCACTGCATTCTAGTTGTGGTNNN</td></tr>
<tr><td>17</td><td>6xCGG</td><td>ACCA</td><td>CGGCGGCGGCGGCGGCGG</td><td>18</td><td>384</td></tr>
<tr><td colspan="6">NNNGATCACTCTCGGCATGGACGAGCTGTACAAGTAAAGCACCAGGCCGCGACTCTAGAT
CATAATCAGCCACCAGACTTCGGCGGCGGCGGCGGCGGACAGCTGGTACCACATTTGTAG</td></tr>
</table>

<table>
<tr><td colspan="6">AGGTTTTACTTGCTTTAAAAAACCTCCCACACCTCCCCCTGAACCTGAAACATAAAGCGAC
TCTAGATCATAATCAGCCATACCACATTTGTAGAGGTTTTACTTGCTTTAAAAAACCTCCC
ACACCTCCCCCTGAACCTGAAACATAAAATGAATGCAATTGTTGTTGTTAACTTGTTTATT
GCAGCTTATAATGGTTACAAATAAAGCAATAGCATCACAAATTTCACAAATAAAGCATTT
TTTTCACTGCATTCTAGTTGTGGTNNN</td></tr>
<tr><td>18</td><td>6xCGT</td><td>ATTA</td><td>CGTCGTCGTCGTCGTCGT</td><td>18</td><td>384</td></tr>
<tr><td colspan="6">NNNGATCACTCTCGGCATGGACGAGCTGTACAAGTAAAGCATTAGGCCGCGACTCTAGAT
CATAATCAGCCACCAGACTTCGTCGTCGTCGTCGTCGTACAGCTGGTACCACATTTGTAGA
GGTTTTACTTGCTTTAAAAAACCTCCCACACCTCCCCCTGAACCTGAAACATAAAGCGACT
CTAGATCATAATCAGCCATACCACATTTGTAGAGGTTTTACTTGCTTTAAAAAACCTCCCA
CACCTCCCCCTGAACCTGAAACATAAAATGAATGCAATTGTTGTTGTTAACTTGTTTATTG
CAGCTTATAATGGTTACAAATAAAGCAATAGCATCACAAATTTCACAAATAAAGCATTTTT
TTCACTGCATTCTAGTTGTGGTNNN</td></tr>
<tr><td>19</td><td>6xCTA</td><td>GGTG</td><td>CTACTACTACTACTACTA</td><td>18</td><td>384</td></tr>
<tr><td colspan="6">NNNGATCACTCTCGGCATGGACGAGCTGTACAAGTAAAGCGGTGGGCCGCGACTCTAGAT
CATAATCAGCCACCAGACTTCTACTACTACTACTACTAACAGCTGGTACCACATTTGTAGA
GGTTTTACTTGCTTTAAAAAACCTCCCACACCTCCCCCTGAACCTGAAACATAAAGCGACT
CTAGATCATAATCAGCCATACCACATTTGTAGAGGTTTTACTTGCTTTAAAAAACCTCCCA
CACCTCCCCCTGAACCTGAAACATAAAATGAATGCAATTGTTGTTGTTAACTTGTTTATTG
CAGCTTATAATGGTTACAAATAAAGCAATAGCATCACAAATTTCACAAATAAAGCATTTTT
TTCACTGCATTCTAGTTGTGGTNNN</td></tr>
<tr><td>20</td><td>6xCTC</td><td>TTGC</td><td>CTCCTCCTCCTCCTCCTC</td><td>18</td><td>384</td></tr>
<tr><td colspan="6">NNNGATCACTCTCGGCATGGACGAGCTGTACAAGTAAAGCTTGCGGCCGCGACTCTAGAT
CATAATCAGCCACCAGACTTCTCCTCCTCCTCCTCCTCACAGCTGGTACCACATTTGTAGA
GGTTTTACTTGCTTTAAAAAACCTCCCACACCTCCCCCTGAACCTGAAACATAAAGCGACT
CTAGATCATAATCAGCCATACCACATTTGTAGAGGTTTTACTTGCTTTAAAAAACCTCCCA
CACCTCCCCCTGAACCTGAAACATAAAATGAATGCAATTGTTGTTGTTAACTTGTTTATTG
CAGCTTATAATGGTTACAAATAAAGCAATAGCATCACAAATTTCACAAATAAAGCATTTTT
TTCACTGCATTCTAGTTGTGGTNNN</td></tr>
<tr><td>21</td><td>6xCTG</td><td>ATAA</td><td>CTGCTGCTGCTGCTGCTG</td><td>18</td><td>384</td></tr>
<tr><td colspan="6">NNNGATCACTCTCGGCATGGACGAGCTGTACAAGTAAAGCATAAGGCCGCGACTCTAGAT
CATAATCAGCCACCAGACTTCTGCTGCTGCTGCTGCTGACAGCTGGTACCACATTTGTAGA
GGTTTTACTTGCTTTAAAAAACCTCCCACACCTCCCCCTGAACCTGAAACATAAAGCGACT
CTAGATCATAATCAGCCATACCACATTTGTAGAGGTTTTACTTGCTTTAAAAAACCTCCCA
CACCTCCCCCTGAACCTGAAACATAAAATGAATGCAATTGTTGTTGTTAACTTGTTTATTG
CAGCTTATAATGGTTACAAATAAAGCAATAGCATCACAAATTTCACAAATAAAGCATTTTT
TTCACTGCATTCTAGTTGTGGTNNN</td></tr>
<tr><td>22</td><td>6xCTT</td><td>GTAT</td><td>CTTCTTCTTCTTCTTCTT</td><td>18</td><td>384</td></tr>
<tr><td colspan="6">NNNGATCACTCTCGGCATGGACGAGCTGTACAAGTAAAGCGTATGGCCGCGACTCTAGAT
CATAATCAGCCACCAGACTTCTTCTTCTTCTTCTTCTTACAGCTGGTACCACATTTGTAGAG
GTTTTACTTGCTTTAAAAAACCTCCCACACCTCCCCCTGAACCTGAAACATAAAGCGACTC
TAGATCATAATCAGCCATACCACATTTGTAGAGGTTTTACTTGCTTTAAAAAACCTCCCAC
ACCTCCCCCTGAACCTGAAACATAAAATGAATGCAATTGTTGTTGTTAACTTGTTTATTGC
AGCTTATAATGGTTACAAATAAAGCAATAGCATCACAAATTTCACAAATAAAGCATTTTTT
TCACTGCATTCTAGTTGTGGTNNN</td></tr>
</table>

<table>
<tr><td>23</td><td>6xGAC</td><td>TACG</td><td>GACGACGACGACGACGAC</td><td>18</td><td>384</td></tr>
<tr><td colspan="6">NNNGATCACTCTCGGCATGGACGAGCTGTACAAGTAAAGCTACGGGCCGCGACTCTAGAT
CATAATCAGCCACCAGACTTGACGACGACGACGACGACACAGCTGGTACCACATTTGTAG
AGGTTTTACTTGCTTTAAAAAACCTCCCACACCTCCCCCTGAACCTGAAACATAAAGCGAC
TCTAGATCATAATCAGCCATACCACATTTGTAGAGGTTTTACTTGCTTTAAAAAACCTCCC
ACACCTCCCCCTGAACCTGAAACATAAAATGAATGCAATTGTTGTTGTTAACTTGTTTATT
GCAGCTTATAATGGTTACAAATAAAGCAATAGCATCACAAATTTCACAAATAAAGCATTT
TTTTCACTGCATTCTAGTTGTGGTNNN</td></tr>
<tr><td>24</td><td>6xGCA</td><td>ACGT</td><td>GCAGCAGCAGCAGCAGCA</td><td>18</td><td>384</td></tr>
<tr><td colspan="6">NNNGATCACTCTCGGCATGGACGAGCTGTACAAGTAAAGCACGTGGCCGCGACTCTAGAT
CATAATCAGCCACCAGACTTGCAGCAGCAGCAGCAGCAACAGCTGGTACCACATTTGTAG
AGGTTTTACTTGCTTTAAAAAACCTCCCACACCTCCCCCTGAACCTGAAACATAAAGCGAC
TCTAGATCATAATCAGCCATACCACATTTGTAGAGGTTTTACTTGCTTTAAAAAACCTCCC
ACACCTCCCCCTGAACCTGAAACATAAAATGAATGCAATTGTTGTTGTTAACTTGTTTATT
GCAGCTTATAATGGTTACAAATAAAGCAATAGCATCACAAATTTCACAAATAAAGCATTT
TTTTCACTGCATTCTAGTTGTGGTNNN</td></tr>
<tr><td>25</td><td>6xGCC</td><td>GCGT</td><td>GCCGCCGCCGCCGCCGCC</td><td>18</td><td>384</td></tr>
<tr><td colspan="6">NNNGATCACTCTCGGCATGGACGAGCTGTACAAGTAAAGCGCGTGGCCGCGACTCTAGAT
CATAATCAGCCACCAGACTTGCCGCCGCCGCCGCCGCCACAGCTGGTACCACATTTGTAG
AGGTTTTACTTGCTTTAAAAAACCTCCCACACCTCCCCCTGAACCTGAAACATAAAGCGAC
TCTAGATCATAATCAGCCATACCACATTTGTAGAGGTTTTACTTGCTTTAAAAAACCTCCC
ACACCTCCCCCTGAACCTGAAACATAAAATGAATGCAATTGTTGTTGTTAACTTGTTTATT
GCAGCTTATAATGGTTACAAATAAAGCAATAGCATCACAAATTTCACAAATAAAGCATTT
TTTTCACTGCATTCTAGTTGTGGTNNN</td></tr>
<tr><td>26</td><td>6xGCG</td><td>CCGG</td><td>GCGGCGGCGGCGGCGGCG</td><td>18</td><td>384</td></tr>
<tr><td colspan="6">NNNGATCACTCTCGGCATGGACGAGCTGTACAAGTAAAGCCCGGGGCCGCGACTCTAGAT
CATAATCAGCCACCAGACTTGCGGCGGCGGCGGCGGCGACAGCTGGTACCACATTTGTAG
AGGTTTTACTTGCTTTAAAAAACCTCCCACACCTCCCCCTGAACCTGAAACATAAAGCGAC
TCTAGATCATAATCAGCCATACCACATTTGTAGAGGTTTTACTTGCTTTAAAAAACCTCCC
ACACCTCCCCCTGAACCTGAAACATAAAATGAATGCAATTGTTGTTGTTAACTTGTTTATT
GCAGCTTATAATGGTTACAAATAAAGCAATAGCATCACAAATTTCACAAATAAAGCATTT
TTTTCACTGCATTCTAGTTGTGGTNNN</td></tr>
<tr><td>27</td><td>6xGCT</td><td>GGTT</td><td>GCTGCTGCTGCTGCTGCT</td><td>18</td><td>384</td></tr>
<tr><td colspan="6">NNNGATCACTCTCGGCATGGACGAGCTGTACAAGTAAAGCGGTTGGCCGCGACTCTAGAT
CATAATCAGCCACCAGACTTGCTGCTGCTGCTGCTGCTACAGCTGGTACCACATTTGTAGA
GGTTTTACTTGCTTTAAAAAACCTCCCACACCTCCCCCTGAACCTGAAACATAAAGCGACT
CTAGATCATAATCAGCCATACCACATTTGTAGAGGTTTTACTTGCTTTAAAAAACCTCCCA
CACCTCCCCCTGAACCTGAAACATAAAATGAATGCAATTGTTGTTGTTAACTTGTTTATTG
CAGCTTATAATGGTTACAAATAAAGCAATAGCATCACAAATTTCACAAATAAAGCATTTTT
TTCACTGCATTCTAGTTGTGGTNNN</td></tr>
<tr><td>28</td><td>6xGGC</td><td>TAAT</td><td>GGCGGCGGCGGCGGCGGC</td><td>18</td><td>384</td></tr>
<tr><td colspan="6">NNNGATCACTCTCGGCATGGACGAGCTGTACAAGTAAAGCTAATGGCCGCGACTCTAGAT
CATAATCAGCCACCAGACTTGGCGGCGGCGGCGGCGGCACAGCTGGTACCACATTTGTAG
AGGTTTTACTTGCTTTAAAAAACCTCCCACACCTCCCCCTGAACCTGAAACATAAAGCGAC
TCTAGATCATAATCAGCCATACCACATTTGTAGAGGTTTTACTTGCTTTAAAAAACCTCCC</td></tr>
</table>

<table>
<tr><td colspan="6">ACACCTCCCCCTGAACCTGAAACATAAAATGAATGCAATTGTTGTTGTTAACTTGTTTATT
GCAGCTTATAATGGTTACAAATAAAGCAATAGCATCACAAATTTCACAAATAAAGCATTT
TTTTCACTGCATTCTAGTTGTGGTNNN</td></tr>
<tr><td>29</td><td>6xGTC</td><td>CATA</td><td>GTCGTCGTCGTCGTCGTC</td><td>18</td><td>384</td></tr>
<tr><td colspan="6">NNNGATCACTCTCGGCATGGACGAGCTGTACAAGTAAAGCCATAGGCCGCGACTCTAGAT
CATAATCAGCCACCAGACTTGTCGTCGTCGTCGTCGTCACAGCTGGTACCACATTTGTAGA
GGTTTTACTTGCTTTAAAAAACCTCCCACACCTCCCCCTGAACCTGAAACATAAAGCGACT
CTAGATCATAATCAGCCATACCACATTTGTAGAGGTTTTACTTGCTTTAAAAAACCTCCCA
CACCTCCCCCTGAACCTGAAACATAAAATGAATGCAATTGTTGTTGTTAACTTGTTTATTG
CAGCTTATAATGGTTACAAATAAAGCAATAGCATCACAAATTTCACAAATAAAGCATTTTT
TTCACTGCATTCTAGTTGTGGTNNN</td></tr>
<tr><td>30</td><td>6xTAC</td><td>AGCC</td><td>TACTACTACTACTACTAC</td><td>18</td><td>384</td></tr>
<tr><td colspan="6">NNNGATCACTCTCGGCATGGACGAGCTGTACAAGTAAAGCAGCCGGCCGCGACTCTAGAT
CATAATCAGCCACCAGACTTTACTACTACTACTACTACACAGCTGGTACCACATTTGTAGA
GGTTTTACTTGCTTTAAAAAACCTCCCACACCTCCCCCTGAACCTGAAACATAAAGCGACT
CTAGATCATAATCAGCCATACCACATTTGTAGAGGTTTTACTTGCTTTAAAAAACCTCCCA
CACCTCCCCCTGAACCTGAAACATAAAATGAATGCAATTGTTGTTGTTAACTTGTTTATTG
CAGCTTATAATGGTTACAAATAAAGCAATAGCATCACAAATTTCACAAATAAAGCATTTTT
TTCACTGCATTCTAGTTGTGGTNNN</td></tr>
<tr><td>31</td><td>6xTCA</td><td>TATG</td><td>TCATCATCATCATCATCA</td><td>18</td><td>384</td></tr>
<tr><td colspan="6">NNNGATCACTCTCGGCATGGACGAGCTGTACAAGTAAAGCTATGGGCCGCGACTCTAGAT
CATAATCAGCCACCAGACTTTCATCATCATCATCATCAACAGCTGGTACCACATTTGTAGA
GGTTTTACTTGCTTTAAAAAACCTCCCACACCTCCCCCTGAACCTGAAACATAAAGCGACT
CTAGATCATAATCAGCCATACCACATTTGTAGAGGTTTTACTTGCTTTAAAAAACCTCCCA
CACCTCCCCCTGAACCTGAAACATAAAATGAATGCAATTGTTGTTGTTAACTTGTTTATTG
CAGCTTATAATGGTTACAAATAAAGCAATAGCATCACAAATTTCACAAATAAAGCATTTTT
TTCACTGCATTCTAGTTGTGGTNNN</td></tr>
<tr><td>32</td><td>6xTCC</td><td>CAAT</td><td>TCCTCCTCCTCCTCCTCC</td><td>18</td><td>384</td></tr>
<tr><td colspan="6">NNNGATCACTCTCGGCATGGACGAGCTGTACAAGTAAAGCCAATGGCCGCGACTCTAGAT
CATAATCAGCCACCAGACTTTCCTCCTCCTCCTCCTCCACAGCTGGTACCACATTTGTAGA
GGTTTTACTTGCTTTAAAAAACCTCCCACACCTCCCCCTGAACCTGAAACATAAAGCGACT
CTAGATCATAATCAGCCATACCACATTTGTAGAGGTTTTACTTGCTTTAAAAAACCTCCCA
CACCTCCCCCTGAACCTGAAACATAAAATGAATGCAATTGTTGTTGTTAACTTGTTTATTG
CAGCTTATAATGGTTACAAATAAAGCAATAGCATCACAAATTTCACAAATAAAGCATTTTT
TTCACTGCATTCTAGTTGTGGTNNN</td></tr>
<tr><td>33</td><td>6xTCG</td><td>ATGC</td><td>TCGTCGTCGTCGTCGTCG</td><td>18</td><td>384</td></tr>
<tr><td colspan="6">NNNGATCACTCTCGGCATGGACGAGCTGTACAAGTAAAGCATGCGGCCGCGACTCTAGAT
CATAATCAGCCACCAGACTTTCGTCGTCGTCGTCGTCGACAGCTGGTACCACATTTGTAGA
GGTTTTACTTGCTTTAAAAAACCTCCCACACCTCCCCCTGAACCTGAAACATAAAGCGACT
CTAGATCATAATCAGCCATACCACATTTGTAGAGGTTTTACTTGCTTTAAAAAACCTCCCA
CACCTCCCCCTGAACCTGAAACATAAAATGAATGCAATTGTTGTTGTTAACTTGTTTATTG
CAGCTTATAATGGTTACAAATAAAGCAATAGCATCACAAATTTCACAAATAAAGCATTTTT
TTCACTGCATTCTAGTTGTGGTNNN</td></tr>
<tr><td>34</td><td>6xTCT</td><td>CGGT</td><td>TCTTCTTCTTCTTCTTCT</td><td>18</td><td>384</td></tr>
</table>

NNNGATCACTCTCGGCATGGACGAGCTGTACAAGTAAAGCCGGTGGCCGCGACTCTAGAT<br>CATAATCAGCCACCAGACTTTCTTCTTCTTCTTCTACAGCTGGTACCACATTTGTAGAG<br>GTTTTACTTGCTTTAAAAAACCTCCCACACCTCCCCCTGAACCTGAAACATAAAGCGACTC<br>TAGATCATAATCAGCCATACCACATTTGTAGAGGTTTTACTTGCTTTAAAAAACCTCCCAC<br>ACCTCCCCCTGAACCTGAAACATAAAATGAATGCAATTGTTGTTGTTAACTTGTTTATTGC<br>AGCTTATAATGGTTACAAATAAAGCAATAGCATCACAAATTTCACAAATAAAGCATTTTTT<br>TCACTGCATTCTAGTTGTGGTNNN					
35	6xTGC	TACC	TGCTGCTGCTGCTGCTGC	18	384
NNNGATCACTCTCGGCATGGACGAGCTGTACAAGTAAAGCTACCGGCCGCGACTCTAGAT<br>CATAATCAGCCACCAGACTTTGCTGCTGCTGCTGCTGCACAGCTGGTACCACATTTGTAGA<br>GGTTTTACTTGCTTTAAAAAACCTCCCACACCTCCCCCTGAACCTGAAACATAAAGCGACT<br>CTAGATCATAATCAGCCATACCACATTTGTAGAGGTTTTACTTGCTTTAAAAAACCTCCCA<br>CACCTCCCCCTGAACCTGAAACATAAAATGAATGCAATTGTTGTTGTTAACTTGTTTATTG<br>CAGCTTATAATGGTTACAAATAAAGCAATAGCATCACAAATTTCACAAATAAAGCATTTTT<br>TTCACTGCATTCTAGTTGTGGTNNN					
36	6XTTC	CCGC	TTCTTCTTCTTCTTCTTC	18	384
NNNGATCACTCTCGGCATGGACGAGCTGTACAAGTAAAGCCCGCGGCCGCGACTCTAGAT<br>CATAATCAGCCACCAGACTTTTCTTCTTCTTCTTCTTCACAGCTGGTACCACATTTGTAGAG<br>GTTTTACTTGCTTTAAAAAACCTCCCACACCTCCCCCTGAACCTGAAACATAAAGCGACTC<br>TAGATCATAATCAGCCATACCACATTTGTAGAGGTTTTACTTGCTTTAAAAAACCTCCCAC<br>ACCTCCCCCTGAACCTGAAACATAAAATGAATGCAATTGTTGTTGTTAACTTGTTTATTGC<br>AGCTTATAATGGTTACAAATAAAGCAATAGCATCACAAATTTCACAAATAAAGCATTTTTT<br>TCACTGCATTCTAGTTGTGGTNNN					
37	JAK2	GTGT	TGGGAATGGCCTGCCT	16	382
NNNGATCACTCTCGGCATGGACGAGCTGTACAAGTAAAGCGTGTGGCCGCGACTCTAGAT<br>CATAATCAGCCACCAGACTTTGGGAATGGCCTGCCTACAGCTGGTACCACATTTGTAGAG<br>GTTTTACTTGCTTTAAAAAACCTCCCACACCTCCCCCTGAACCTGAAACATAAAGCGACTC<br>TAGATCATAATCAGCCATACCACATTTGTAGAGGTTTTACTTGCTTTAAAAAACCTCCCAC<br>ACCTCCCCCTGAACCTGAAACATAAAATGAATGCAATTGTTGTTGTTAACTTGTTTATTGC<br>AGCTTATAATGGTTACAAATAAAGCAATAGCATCACAAATTTCACAAATAAAGCATTTTTT<br>TCACTGCATTCTAGTTGTGGTNNN					
38	SIRT6	TAGT	GCCGTACGCGGACAAGGG	18	384
NNNGATCACTCTCGGCATGGACGAGCTGTACAAGTAAAGCTAGTGGCCGCGACTCTAGAT<br>CATAATCAGCCACCAGACTTGCCGTACGCGGACAAGGGACAGCTGGTACCACATTTGTAG<br>AGGTTTTACTTGCTTTAAAAAACCTCCCACACCTCCCCCTGAACCTGAAACATAAAGCGAC<br>TCTAGATCATAATCAGCCATACCACATTTGTAGAGGTTTTACTTGCTTTAAAAAACCTCCC<br>ACACCTCCCCCTGAACCTGAAACATAAAATGAATGCAATTGTTGTTGTTAACTTGTTTATT<br>GCAGCTTATAATGGTTACAAATAAAGCAATAGCATCACAAATTTCACAAATAAAGCATTT<br>TTTTCACTGCATTCTAGTTGTGGTNNN					

Supplementary Table 2. Summary of reported C/G to T/A conversions on human mtDNA.

**Base Mutations**	**Number**
GC>GT	95
TC>TT	96
AC>AT	91
CC>CT	78
Total	360

Data from MITOMAP database (updated on July 15, 2023).

Supplementary Table 3. Plasmid suite containing m.A8344G mutation.

<table>
<tr><th>ID</th><th>Spacer Name</th><th>Barcode</th><th>Spacer Sequence</th><th>Spacer Length</th><th>Amplicon Length</th></tr>
<tr><td>1</td><td>A8344G-1</td><td>ATTG</td><td>AAGAGAGCCAACACC</td><td>15</td><td>381</td></tr>
<tr><td colspan="6">NNNGATCACTCTCGGCATGGACGAGCTGTACAAGTAAAGCATTGGGCCGCGACTCTAGAT
CATAATCAGCCACCAGACTTAAGAGAGCCAACACCACAGCTGGTACCACATTTGTAGAGG
TTTTACTTGCTTTAAAAAACCTCCCACACCTCCCCCTGAACCTGAAACATAAAGCGACTCT
AGATCATAATCAGCCATACCACATTTGTAGAGGTTTTACTTGCTTTAAAAAACCTCCCACA
CCTCCCCCTGAACCTGAAACATAAAATGAATGCAATTGTTGTTGTTAACTTGTTTATTGCA
GCTTATAATGGTTACAAATAAAGCAATAGCATCACAAATTTCACAAATAAAGCATTTTTTT
CACTGCATTCTAGTTGTGGTNNN</td></tr>
<tr><td>2</td><td>A8344G-2</td><td>TGGC</td><td>AGAGAGCCAACACCT</td><td>15</td><td>381</td></tr>
<tr><td colspan="6">NNNGATCACTCTCGGCATGGACGAGCTGTACAAGTAAAGCTGGCGGCCGCGACTCTAGAT
CATAATCAGCCACCAGACTTAGAGAGCCAACACCTACAGCTGGTACCACATTTGTAGAGG
TTTTACTTGCTTTAAAAAACCTCCCACACCTCCCCCTGAACCTGAAACATAAAGCGACTCT
AGATCATAATCAGCCATACCACATTTGTAGAGGTTTTACTTGCTTTAAAAAACCTCCCACA
CCTCCCCCTGAACCTGAAACATAAAATGAATGCAATTGTTGTTGTTAACTTGTTTATTGCA
GCTTATAATGGTTACAAATAAAGCAATAGCATCACAAATTTCACAAATAAAGCATTTTTTT
CACTGCATTCTAGTTGTGGTNNN</td></tr>
<tr><td>3</td><td>A8344G-3</td><td>GCGG</td><td>AGAGAGCCAACACC</td><td>14</td><td>380</td></tr>
<tr><td colspan="6">NNNGATCACTCTCGGCATGGACGAGCTGTACAAGTAAAGCGCGGGGCCGCGACTCTAGAT
CATAATCAGCCACCAGACTTAGAGAGCCAACACCACAGCTGGTACCACATTTGTAGAGGT
TTTACTTGCTTTAAAAAACCTCCCACACCTCCCCCTGAACCTGAAACATAAAGCGACTCTA
GATCATAATCAGCCATACCACATTTGTAGAGGTTTTACTTGCTTTAAAAAACCTCCCACAC
CTCCCCCTGAACCTGAAACATAAAATGAATGCAATTGTTGTTGTTAACTTGTTTATTGCAG
CTTATAATGGTTACAAATAAAGCAATAGCATCACAAATTTCACAAATAAAGCATTTTTTTC
ACTGCATTCTAGTTGTGGTNNN</td></tr>
<tr><td>4</td><td>A8344G-4</td><td>CCTA</td><td>GAGAGCCAACACCT</td><td>14</td><td>380</td></tr>
<tr><td colspan="6">NNNGATCACTCTCGGCATGGACGAGCTGTACAAGTAAAGCCCTAGGCCGCGACTCTAGAT
CATAATCAGCCACCAGACTTGAGAGCCAACACCTACAGCTGGTACCACATTTGTAGAGGT
TTTACTTGCTTTAAAAAACCTCCCACACCTCCCCCTGAACCTGAAACATAAAGCGACTCTA
GATCATAATCAGCCATACCACATTTGTAGAGGTTTTACTTGCTTTAAAAAACCTCCCACAC
CTCCCCCTGAACCTGAAACATAAAATGAATGCAATTGTTGTTGTTAACTTGTTTATTGCAG
CTTATAATGGTTACAAATAAAGCAATAGCATCACAAATTTCACAAATAAAGCATTTTTTTC
ACTGCATTCTAGTTGTGGTNNN</td></tr>
<tr><td>5</td><td>A8344G-5</td><td>CGCG</td><td>AGAGCCAACACCTC</td><td>14</td><td>380</td></tr>
<tr><td colspan="6">NNNGATCACTCTCGGCATGGACGAGCTGTACAAGTAAAGCCGCGGGCCGCGACTCTAGAT
CATAATCAGCCACCAGACTTAGAGCCAACACCTCACAGCTGGTACCACATTTGTAGAGGT
TTTACTTGCTTTAAAAAACCTCCCACACCTCCCCCTGAACCTGAAACATAAAGCGACTCTA
GATCATAATCAGCCATACCACATTTGTAGAGGTTTTACTTGCTTTAAAAAACCTCCCACAC
CTCCCCCTGAACCTGAAACATAAAATGAATGCAATTGTTGTTGTTAACTTGTTTATTGCAG
CTTATAATGGTTACAAATAAAGCAATAGCATCACAAATTTCACAAATAAAGCATTTTTTTC
ACTGCATTCTAGTTGTGGTNNN</td></tr>
<tr><td>6</td><td>A8344G-6</td><td>GGCT</td><td>AGAGCCAACACC</td><td>12</td><td>378</td></tr>
</table>

<table>
<tr><td colspan="6">NNNGATCACTCTCGGCATGGACGAGCTGTACAAGTAAAGCGGCTGGCCGCGACTCTAGATCATAATCAGCCACCAGACTTAGAGCCAACACCACAGCTGGTACCACATTTGTAGAGGTTTTACTTGCTTTAAAAAACCTCCCACACCTCCCCCTGAACCTGAAACATAAAGCGACTCTAGATCATAATCAGCCATACCACATTTGTAGAGGTTTTACTTGCTTTAAAAAACCTCCCACACCTCCCCCTGAACCTGAAACATAAAATGAATGCAATTGTTGTTGTTAACTTGTTTATTGCAGCTTATAATGGTTACAAATAAAGCAATAGCATCACAAATTTCACAAATAAAGCATTTTTTTCACTGCATTCTAGTTGTGGTNNN</td></tr>
<tr><td>7</td><td>A8344G-7</td><td>CCAG</td><td>AGAGCCAACAC</td><td>11</td><td>377</td></tr>
<tr><td colspan="6">NNNGATCACTCTCGGCATGGACGAGCTGTACAAGTAAAGCCCAGGGCCGCGACTCTAGATCATAATCAGCCACCAGACTTAGAGCCAACACACAGCTGGTACCACATTTGTAGAGGTTTTACTTGCTTTAAAAAACCTCCCACACCTCCCCCTGAACCTGAAACATAAAGCGACTCTAGATCATAATCAGCCATACCACATTTGTAGAGGTTTTACTTGCTTTAAAAAACCTCCCACACCTCCCCCTGAACCTGAAACATAAAATGAATGCAATTGTTGTTGTTAACTTGTTTATTGCAGCTTATAATGGTTACAAATAAAGCAATAGCATCACAAATTTCACAAATAAAGCATTTTTTTCACTGCATTCTAGTTGTGGTNNN</td></tr>
<tr><td>8</td><td>A8344G-8</td><td>TGTG</td><td>AGAGCCAACA</td><td>10</td><td>376</td></tr>
<tr><td colspan="6">NNNGATCACTCTCGGCATGGACGAGCTGTACAAGTAAAGCTGTGGGCCGCGACTCTAGATCATAATCAGCCACCAGACTTAGAGCCAACAACAGCTGGTACCACATTTGTAGAGGTTTTACTTGCTTTAAAAAACCTCCCACACCTCCCCCTGAACCTGAAACATAAAGCGACTCTAGATCATAATCAGCCATACCACATTTGTAGAGGTTTTACTTGCTTTAAAAAACCTCCCACACCTCCCCCTGAACCTGAAACATAAAATGAATGCAATTGTTGTTGTTAACTTGTTTATTGCAGCTTATAATGGTTACAAATAAAGCAATAGCATCACAAATTTCACAAATAAAGCATTTTTTTCACTGCATTCTAGTTGTGGTNNN</td></tr>
<tr><td>9</td><td>A8344G-9</td><td>CGCA</td><td>GAGAGCCAACA</td><td>11</td><td>377</td></tr>
<tr><td colspan="6">NNNGATCACTCTCGGCATGGACGAGCTGTACAAGTAAAGCCGCAGGCCGCGACTCTAGATCATAATCAGCCACCAGACTTGAGAGCCAACAACAGCTGGTACCACATTTGTAGAGGTTTTACTTGCTTTAAAAAACCTCCCACACCTCCCCCTGAACCTGAAACATAAAGCGACTCTAGATCATAATCAGCCATACCACATTTGTAGAGGTTTTACTTGCTTTAAAAAACCTCCCACACCTCCCCCTGAACCTGAAACATAAAATGAATGCAATTGTTGTTGTTAACTTGTTTATTGCAGCTTATAATGGTTACAAATAAAGCAATAGCATCACAAATTTCACAAATAAAGCATTTTTTTCACTGCATTCTAGTTGTGGTNNN</td></tr>
<tr><td>10</td><td>A8344G-10</td><td>CGGC</td><td>AGAGAGCCAACA</td><td>12</td><td>378</td></tr>
<tr><td colspan="6">NNNGATCACTCTCGGCATGGACGAGCTGTACAAGTAAAGCCGGCGGCCGCGACTCTAGATCATAATCAGCCACCAGACTTAGAGAGCCAACAACAGCTGGTACCACATTTGTAGAGGTTTTACTTGCTTTAAAAAACCTCCCACACCTCCCCCTGAACCTGAAACATAAAGCGACTCTAGATCATAATCAGCCATACCACATTTGTAGAGGTTTTACTTGCTTTAAAAAACCTCCCACACCTCCCCCTGAACCTGAAACATAAAATGAATGCAATTGTTGTTGTTAACTTGTTTATTGCAGCTTATAATGGTTACAAATAAAGCAATAGCATCACAAATTTCACAAATAAAGCATTTTTTTCACTGCATTCTAGTTGTGGTNNN</td></tr>
</table>

Supplementary Table 4. Primers.

RVD Sanger Sequencing Primers	Sequence
RVD seq Fwd	TGACCGCAGTGGAGGCAGTG
RVD seq Rev	TTCACTGCATCCAGCGCAGG
m.A8344G Sanger Sequencing Primers	Sequence
m.A8344G seq Fwd	AGCAAACCACAGTTTCATGCC
m.A8344G seq Rev	TAATCACTGTGCCCGCTCAT
Amplicon Sequencing Primers	Sequence
i5-spacer-lib-Fwd1	ACACTCTTTCCCTACACGACGCTCTTCCGATCTTCAGATCACTCTCGGCATGGACG
i5-spacer-lib-Fwd2	ACACTCTTTCCCTACACGACGCTCTTCCGATCTCAAGATCACTCTCGGCATGGACG
i5-spacer-lib-Fwd3	ACACTCTTTCCCTACACGACGCTCTTCCGATCTGTAGATCACTCTCGGCATGGACG
i7-spacer-lib-Rev1	GTGACTGGAGTTCAGACGTGTGCTCTTCCGATCTACCACCACAACTAGAATGCAGTG
i7-spacer-lib-Rev2	GTGACTGGAGTTCAGACGTGTGCTCTTCCGATCTTAGACCACAACTAGAATGCAGTG
i7-spacer-lib-Rev3	GTGACTGGAGTTCAGACGTGTGCTCTTCCGATCTGACACCACAACTAGAATGCAGTG
i5-m.G8313-Fwd1	ACACTCTTTCCCTACACGACGCTCTTCCGATCTGTAGCAAACCACAGTTTCATGCCCA
i5-m.G8313-Fwd2	ACACTCTTTCCCTACACGACGCTCTTCCGATCTAGTGCAAACCACAGTTTCATGCCCA
i5-m.G8313-Fwd3	ACACTCTTTCCCTACACGACGCTCTTCCGATCTTCGGCAAACCACAGTTTCATGCCCA
i7-m.G8313-Rev1	GTGACTGGAGTTCAGACGTGTGCTCTTCCGATCTGTATATGGTGGGCCATACGGTAGTA
i7-m.G8313-Rev2	GTGACTGGAGTTCAGACGTGTGCTCTTCCGATCTAGTTATGGTGGGCCATACGGTAGTA
i7-m.G8313-Rev3	GTGACTGGAGTTCAGACGTGTGCTCTTCCGATCTTCGTATGGTGGGCCATACGGTAGTA
i5-n.JAK2-Fwd1	ACACTCTTTCCCTACACGACGCTCTTCCGATCTGTAGCTAGGATTACAGGTGTGAGAC
i5-n.JAK2-Fwd2	ACACTCTTTCCCTACACGACGCTCTTCCGATCTAGTGCTAGGATTACAGGTGTGAGAC
i5-n.JAK2-Fwd3	ACACTCTTTCCCTACACGACGCTCTTCCGATCTTCGGCTAGGATTACAGGTGTGAGAC
i7-n.JAK2-Rev1	GTGACTGGAGTTCAGACGTGTGCTCTTCCGATCTGTAGATACCTAGCCCAAGGCAGA

i7-n.JAK2-Rev2	GTGACTGGAGTTCAGACGTGTGCTCTTCCGATCTAGTGATA CCTAGCCCAAGGCAGA
i7-n.JAK2-Rev3	GTGACTGGAGTTCAGACGTGTGCTCTTCCGATCTTCGGATA CCTAGCCCAAGGCAGA
Whole mtDNA Sequencing Primers	Sequence
mtDNA-Fwd1	CCTCACCACCTCTTGCTCAG
mtDNA-Rev1	TAGGGGATTTAGCGGGGTGA
mtDNA-Fwd2	CAGCCCATGACCCCTAACAG
mtDNA-Rev2	AGGGCCCTGTTCAACTAAGC

Supplementary Table 5. C/G to T/A SNPs on HEK293FT mtDNA.

**ID**	**SNPs**
1	chrMT_1412_G
2	chrMT_1414_C
3	chrMT_4429_G
4	chrMT_4707_C
5	chrMT_5339_C
6	chrMT_7028_C
7	chrMT_9477_G
8	chrMT_11719_G
9	chrMT_12372_G
10	chrMT_12801_C
11	chrMT_13889_G
12	chrMT_14766_C
13	chrMT_15466_G
14	chrMT_16192_C
15	chrMT_16256_C
16	chrMT_16270_C

Supplementary Note. Amino acid sequences used in this study.

Amino acid sequences are annotated as: red for MTS, purple for NLS, yellow for Flag tag, italics for *linker*, green for NTD/CTD, underlined for RVD, cyan for UGI, orange font for split halves of DddA or its homologs and green for DddI_A.

Mitochondrial Targeting Signal (MTS):

MLGFVGRVAAAPASGALRRLTPSASLPPAQLLLRAAPTAVHPVRDYAAQTSESGGGGSPG

Nuclear Localization Signal (NLS):

PKKKRKV

Flag:

DYKDDDDK

Linker:

AAA

GS

G*GS*

SGGS

N-terminal domain (NTD):

VDLRTLGYSQQQQEKIKPKVRSTVAQHHEALVGHGFTHAHIVALSQHPAALGTVAVKYQ
DMIAALPEATHEAIVGVGKQWSGARALEALLTVAGELRGPPLQLDTGQLLKIAKRGGVT
AVEAVHAWRNALTGAPLN

C-terminal domain (CTD):

ALTNDHLVALACLGGRPALDAVKKGL

Uracil Glycosylase Inhibitor (UGI):

TNLSDIIEKETGKQLVIQESILMLPEEVEEVIGNKPESDILVHTAYDESTDENVMLLTSDAP
EYKPWALVIQDSNGENKIKML

MTS-DdCBEs:

MLGFVGRVAAAPASGALRRLTPSASLPPAQLLLRAAPTAVHPVRDYAAQTSESGGGGSPG *AAA*DYKDDDDK*GS*VDLRTLGYSQQQQEKIKPKVRSTVAQHHEALVGHGFTHAHIVALSQ HPAALGTVAVKYQDMIAALPEATHEAIVGVGKRGAGARALEALLTVAGELRGPPLQLDT GQLLKIAKRGGVTAVEAVHAWRNALTGAPLN-TALE-ALTNDHLVALACLGGRPALDAVKKGLG*GS*-Split Halves-*SGGS*TNLSDIIEKETGKQLVIQESILMLPEEVEEVIGNKPESDILVHTAYDESTDENVMLLTS DAPEYKPWALVIQDSNGENKIKML

NLS-DdCBEs:

PKKKRKV*AAA*DYKDDDDK*GS*VDLRTLGYSQQQQEKIKPKVRSTVAQHHEALVGHGFTH AHIVALSQHPAALGTVAVKYQDMIAALPEATHEAIVGVGKRGAGARALEALLTVAGELR GPPLQLDTGQLLKIAKRGGVTAVEAVHAWRNALTGAPLN-TALE-ALTNDHLVALACLGGRPALDAVKKGLG*GS*-Split Halves-*SGGS*TNLSDIIEKETGKQLVIQESILMLPEEVEEVIGNKPESDILVHTAYDESTDENVMLLTS DAPEYKPWALVIQDSNGENKIKML

NLS-DddI$_A$:

PKKKRKV*AAA*DYKDDDDK*GS*-DddI$_A$

G1333C (DddA):

PTPYPNYANAGHVEGQSALFMRDNGISEGLVFHNNPEGTCGFCVNMTETLLPENAKMT
VVPPEGAIPVKRGATGETKVFTGNSNSPKSPTKGGC

G1333N (DddA):

GSYALGPYQISAPQLPAYNGQTVGTFYYVNDAGGLESKVFSSGG

G1397C (DddA):

AIPVKRGATGETKVFTGNSNSPKSPTKGGC

G1397N (DddA):

GSYALGPYQISAPQLPAYNGQTVGTFYYVNDAGGLESKVFSSGGPTPYPNYANAGHVEG
QSALFMRDNGISEGLVFHNNPEGTCGFCVNMTETLLPENAKMTVVPPEG

G1333C (DddA6):

PTPYPNYANAGHVEGQSALFMRDNGISEGLVFHNNPEGTCGFCVNMIETLLPENAKMTV
VPPEGAIPVKRGATGETKVFIGNSNSPKSPTKGGC

G1333N (DddA6):

GSYALGPYQISAPQLPAYNGRTVGTFYYVNDAGGLESKVFISGG

G1397C (DddA6):

AIPVKRGATGETKVFIGNSNSPKSPTKGGC

G1397N (DddA6):

GSYALGPYQISAPQLPAYNGRTVGTFYYVNDAGGLESKVFISGGPTPYPNYANAGHVEG
QSALFMRDNGISEGLVFHNNPEGTCGFCVNMIETLLPENAKMTVVPPEG

G1333C (DddA11):

PTPYPNYVSAGHVEGQSALFMRDNGISEGLVFHNNPKGTCGFCVNMIETLLPENAKMTV
VPPEGAIPVKRGATGETKVFIGNSNSPKSPTKGGC

G1333N (DddA11):

GSYALGPYQISAPQLPAYNGQTVGTFYYVNDAGGLESKVFISGG

G1397C (DddA11):

AIPVKRGATGETKVFIGNSNSPKSPTKGGC

G1397N (DddA11):

GSYALGPYQISAPQLPAYNGQTVGTFYYVNDAGGLESKVFISGGPTPYPNYVSAGHVEG

QSALFMRDNGISEGLVFHNNPKGTCGFCVNMIETLLPENAKMTVVPPEG

G28C (FZY2):

NGDPRYTNYRNNGHVEQKSALYMRENNISNATVYHNNTNGTCGYCNTMTATFLPEGAT
LTVVPPENAVANNSRAIDYVKTYTGTSNDPKISPRYKGN

G28N (FZY2):

MSLPEYDGTTTHGVLVLDDGTQIGFTSG

S100C (FZY2):

RAIDYVKTYTGTSNDPKISPRYKGN

S100N (FZY2):

MSLPEYDGTTTHGVLVLDDGTQIGFTSGNGDPRYTNYRNNGHVEQKSALYMRENNISN
ATVYHNNTNGTCGYCNTMTATFLPEGATLTVVPPENAVANNS

S100N (FZY2 v1.1):

MSLPEYDG**R**TTHGVLVLDDGTQIGFTSGNGDPRYTNYRNNGHVEQKSALYMRENNISN
ATVYHNNTNGTCGYCNTMTATFLPEGATLTVVPPENAVANNS

S100N (FZY2 v1.2):

MSLPEYDGTTTHGVLVLDDGTQIGF**I**SGNGDPRYTNYRNNGHVEQKSALYMRENNISNA
TVYHNNTNGTCGYCNTMTATFLPEGATLTVVPPENAVANNS

S100N (FZY2 v1.3):

MSLPEYDGTTTHGVLVLDDGTQIGFTSGNGDPRYTNY**V**NNGHVEQKSALYMRENNISN
ATVYHNNTNGTCGYCNTMTATFLPEGATLTVVPPENAVANNS

S100N (FZY2 v1.4):

MSLPEYDGTTTHGVLVLDDGTQIGFTSGNGDPRYTNYR**S**NGHVEQKSALYMRENNISNA
TVYHNNTNGTCGYCNTMTATFLPEGATLTVVPPENAVANNS

S100N (FZY2 v1.5):

MSLPEYDGTTTHGVLVLDDGTQIGFTSGNGDPRYTNYRNNGHVEQKSALYMRENNISN
ATVYHNNT**E**GTCGYCNTMTATFLPEGATLTVVPPENAVANNS

S100N (FZY2 v1.6):

MSLPEYDGTTTHGVLVLDDGTQIGFTSGNGDPRYTNYRNNGHVEQKSALYMRENNISN
ATVYHNNTNGTCGYCNTM**I**ATFLPEGATLTVVPPENAVANNS

S100C (FZY2 v1.7):

RAIDYVKTY**I**GTSNDPKISPRYKGN

S193C (WC03):

*GS*YPQYKAQSASHVEGKAALYMRENGINEATVFHNNPNGTCGFCDRQVPALLPKGAKL
TVVPPSNSVANNVRAIPVPKTYIGNSTVPKIK

S193N (WC03):

NANCNQEKPVLPKYDGKTTEGVMVTPDGKQISFKSGNSSTPS

S257C (WC03):

VANNVRAIPVPKTYIGNSTVPKIK

S257N (WC03):

NANCNQEKPVLPKYDGKTTEGVMVTPDGKQISFKSGNSSTPSYPQYKAQSASHVEGKA
ALYMRENGINEATVFHNNPNGTCGFCDRQVPALLPKGAKLTVVPPSNS

G181C (XYI6):

NPNPNYKNYIPASHVEGKSAIYMRENGITSGTIYYNNTDGTCPYCDKMLSTLLEEGSVLE
VIPPINAKAPKPSWVDKPKTYIGNNKVPKPNK

G181N (XYI6):

NMSITDRLAKQKEKQDNTNIIDNRPKLPDYDGKTTHGILVTPNSEHIPFSSG

N247C (XYI6):

AKAPKPSWVDKPKTYIGNNKVPKPNK

N247C (XYI6):

SNMSITDRLAKQKEKQDNTNIIDNRPKLPDYDGKTTHGILVTPNSEHIPFSSGNPNPNYKN
YIPASHVEGKSAIYMRENGITSGTIYYNNTDGTCPYCDKMLSTLLEEGSVLEVIPPIN

G1414C (L9D3):

KVLSNYDSSGHVEGMAALIMRKGRITEAVVMHNHPSGTCHYCNGQVETLLPKNAKLK
VIPPANAKAPTKYWYDQPVDYLGNSNDPKPPS

G1414N (L9D3):

ALLREQFPSMDAVTLPPFDGKTTIGYMFYTDANGQYHVRKLYSGG

N1474C (L9D3):

AKAPTKYWYDQPVDYLGNSNDPKPPS

N1474N (L9D3):

ALLREQFPSMDAVTLPPFDGKTTIGYMFYTDANGQYHVRKLYSGGKVLSNYDSSGHVE
GMAALIMRKGRITEAVVMHNHPSGTCHYCNGQVETLLPKNAKLKVIPPAN

G1794C (HR14):

RPSLPNYIASGHVEGQAAMIMRQQQVQSATVYHDNPNGTCGYCYSQLPTLLPEGAALD
VVPPAGTVPPSNRWHNGGPSFIGNSSEPKPWPR

G1794N (HR14):

GTSSDTIAEMLNSASQPGRTAGVLDIDGELTPLTSG

G1858C (HR14):

TVPPSNRWHNGGPSFIGNSSEPKPWPR

G1858N (HR14):

GTSSDTIAEMLNSASQPGRTAGVLDIDGELTPLTSGRPSLPNYIASGHVEGQAAMIMRQQ
QVQSATVYHDNPNGTCGYCYSQLPTLLPEGAALDVVPPAG

T2113C (Q2L7):

SSLKNYAASGHVEGQAALIMRERGVASARLIIDNPSGICGYCRSQVPTLLPAGATLEVTTP
RGTVPPTARWSNGKTFVGNENDPKPWPR

T2113N (Q2L7):

GKVGKLRFAPKVESAESMLRSLSQEGKTAGVLDINGELIPLVSGT

G2176C (Q2L7):

TVPPTARWSNGKTFVGNENDPKPWPR

G2176N (Q2L7):

GKVGKLRFAPKVESAESMLRSLSQEGKTAGVLDINGELIPLVSGTSSLKNYAASGHVEGQ
AALIMRERGVASARLIIDNPSGICGYCRSQVPTLLPAGATLEVTTPRG

G1427C (XG57):

KVLSNYDATGHVEGKAALIMRNEKITEAVVMHNHPSGTCNYCDKQVETLLPKNATLRVI
PPENAKAPTSYWNDQPTTYRGDGKDPKAPSKK

G1427N (XG57):

AYPSMEGATLPPFDGKTTIGLMFYTDASGQYQVKKLFSG

N1491C (XG57):

AKAPTSYWNDQPTTYRGDGKDPKAPSKK

N1491N (XG57):

AYPSMEGATLPPFDGKTTIGLMFYTDASGQYQVKKLFSGEKVLSNYDATGHVEGKAALI
MRNEKITEAVVMHNHPSGTCNYCDKQVETLLPKNATLRVIPPEN

Spacer Plasmid Left TALE:
LTPEQVVAIASNIGGKQALETVQRLLPVLCQAHGLTPDQVVAIASNIGGKQALETVQRLL
PVLCQAHGLTPDQVVAIASNGGGKQALETVQRLLPVLCQAHGLTPEQVVAIASHDGGKQ
ALETVQRLLPVLCQAHGLTPAQVVAIASNIGGKQALETVQRLLPVLCQAHGLTPDQVVAI
ASNNGGKQALETVQRLLPVLCQAHGLTPEQVVAIASHDGGKQALETVQRLLPVLCQAH
GLTPAQVVAIASHDGGKQALETVQRLLPVLCQAHGLTPAQVVAIASNIGGKQALETVQRL
LPVLCQAHGLTPDQVVAIASHDGGKQALETVQRLLPVLCQAHGLTPAQVVAIASHDGGK
QALETVQRLLPVLCQAHGLTPAQVVAIASNIGGKQALETVQRLLPVLCQAHGLTPDQVV
AIASNNGGKQALETVQRLLPVLCQAHGLTPEQVVAIASNIGGKQALETVQRLLPVLCQA
HGLTPDQVVAIASHDGGKQALETVQRLLPVLCQAHGLTPAQVVAIASNGGGKQALETVQ
RLLPVLCQAHGLTPEQVVAIASNGGGRPALESIVAQLSRPDPALA

Spacer Plasmid Right TALE:
LTPEQVVAIASNNGGKQALETVQRLLPVLCQAHGLTPEQVVAIASNGGGKQALETVQRL
LPVLCQAHGLTPEQVVAIASNNGGKQALETVQRLLPVLCQAHGLTPEQVVAIASNNGGK
QALETVQRLLPVLCQAHGLTPEQVVAIASNGGGKQALETVQRLLPVLCQAHGLTPEQVV
AIASNIGGKQALETVQRLLPVLCQAHGLTPDQVVAIASHDGGKQALETVQRLLPVLCQA
HGLTPAQVVAIASHDGGKQALETVQRLLPVLCQAHGLTPAQVVAIASNIGGKQALETVQ
RLLPVLCQAHGLTPDQVVAIASNNGGKQALETVQRLLPVLCQAHGLTPEQVVAIASHDG
GKQALETVQRLLPVLCQAHGLTPAQVVAIASNGGGKQALETVQRLLPVLCQAHGLTPEQ
VVAIASNNGGKQALETVQRLLPVLCQAHGLTPEQVVAIASNGGGRPALESIVAQLSRPDP
ALA

Human m.G3460 Left TALE:

LTPEQVVAIASNIGGKQALETVQRLLPVLCQAHGLTPDQVVAIASHDGGKQALETVQRLL
PVLCQAHGLTPAQVVAIASNGGGKQALETVQRLLPVLCQAHGLTPEQVVAIASNIGGKQ
ALETVQRLLPVLCQAHGLTPDQVVAIASHDGGKQALETVQRLLPVLCQAHGLTPAQVVA
IASNIGGKQALETVQRLLPVLCQAHGLTPDQVVAIASNIGGKQALETVQRLLPVLCQAHG
LTPDQVVAIASHDGGKQALETVQRLLPVLCQAHGLTPAQVVAIASHDGGKQALETVQRL
LPVLCQAHGLTPAQVVAIASHDGGKQALETVQRLLPVLCQAHGLTPAQVVAIASNGGGK
QALETVQRLLPVLCQAHGLTPEQVVAIASNGGGKQALETVQRLLPVLCQAHGLTPEQVV
AIASHDGGKQALETVQRLLPVLCQAHGLTPAQVVAIASNNGGKQALETVQRLLPVLCQA
HGLTPEQVVAIASHDGGKQALETVQRLLPVLCQAHGLTPAQVVAIASNGGGRPALESIVA
QLSRPDPALA

Human m.G3460 Right TALE:

LTPEQVVAIASNIGGKQALETVQRLLPVLCQAHGLTPDQVVAIASNNGGKQALETVQRLL
PVLCQAHGLTPEQVVAIASNNGGKQALETVQRLLPVLCQAHGLTPEQVVAIASNNGGKQ
ALETVQRLLPVLCQAHGLTPEQVVAIASNNGGKQALETVQRLLPVLCQAHGLTPEQVVA
IASHDGGKQALETVQRLLPVLCQAHGLTPAQVVAIASNGGGKQALETVQRLLPVLCQAH
GLTPEQVVAIASHDGGKQALETVQRLLPVLCQAHGLTPAQVVAIASNGGGKQALETVQR
LLPVLCQAHGLTPEQVVAIASNGGGKQALETVQRLLPVLCQAHGLTPEQVVAIASNGGG
KQALETVQRLLPVLCQAHGLTPEQVVAIASNNGGKQALETVQRLLPVLCQAHGLTPEQV
VAIASNNGGKQALETVQRLLPVLCQAHGLTPEQVVAIASNGGGKQALETVQRLLPVLCQ
AHGLTPEQVVAIASNNGGKQALETVQRLLPVLCQAHGLTPEQVVAIASNIGGKQALETV
QRLLPVLCQAHGLTPDQVVAIASNIGGKQALETVQRLLPVLCQAHGLTPDQVVAIASNN
GGKQALETVQRLLPVLCQAHGLTPEQVVAIASNIGGRPALESIVAQLSRPDPALA

Human m.G3635 Left TALE:

LTPEQVVAIASHDGGKQALETVQRLLPVLCQAHGLTPAQVVAIASHDGGKQALETVQRL
LPVLCQAHGLTPAQVVAIASNGGGKQALETVQRLLPVLCQAHGLTPEQVVAIASNIGGK
QALETVQRLLPVLCQAHGLTPDQVVAIASNGGGKQALETVQRLLPVLCQAHGLTPEQVV
AIASNGGGKQALETVQRLLPVLCQAHGLTPEQVVAIASNGGGKQALETVQRLLPVLCQA
HGLTPEQVVAIASNIGGKQALETVQRLLPVLCQAHGLTPDQVVAIASNGGGKQALETVQ
RLLPVLCQAHGLTPEQVVAIASNGGGKQALETVQRLLPVLCQAHGLTPEQVVAIASHDG
GKQALETVQRLLPVLCQAHGLTPAQVVAIASNGGGKQALETVQRLLPVLCQAHGLTPEQ
VVAIASNIGGKQALETVQRLLPVLCQAHGLTPDQVVAIASNNGGKQALETVQRLLPVLC
QAHGLTPEQVVAIASHDGGKQALETVQRLLPVLCQAHGLTPAQVVAIASHDGGKQALET
VQRLLPVLCQAHGLTPAQVVAIASNIGGRPALESIVAQLSRPDPALA

Human m.G3635 Right TALE:

LTPEQVVAIASHDGGKQALETVQRLLPVLCQAHGLTPAQVVAIASNIGGKQALETVQRLL
PVLCQAHGLTPDQVVAIASNNGGKQALETVQRLLPVLCQAHGLTPEQVVAIASNIGGKQ
ALETVQRLLPVLCQAHGLTPDQVVAIASNNGGKQALETVQRLLPVLCQAHGLTPEQVVA
IASNNGGKQALETVQRLLPVLCQAHGLTPEQVVAIASNIGGKQALETVQRLLPVLCQAH
GLTPDQVVAIASNGGGKQALETVQRLLPVLCQAHGLTPEQVVAIASNGGGKQALETVQR
LLPVLCQAHGLTPEQVVAIASNNGGKQALETVQRLLPVLCQAHGLTPEQVVAIASNIGGK
QALETVQRLLPVLCQAHGLTPDQVVAIASNNGGKQALETVQRLLPVLCQAHGLTPEQVV
AIASNGGGKQALETVQRLLPVLCQAHGLTPEQVVAIASNIGGKQALETVQRLLPVLCQA
HGLTPDQVVAIASNIGGKQALETVQRLLPVLCQAHGLTPDQVVAIASNIGGRPALESIVAQ
LSRPDPALA

Human m.G8313 Left TALE:

LTPEQVVAIASNIGGKQALETVQRLLPVLCQAHGLTPDQVVAIASNNGGKQALETVQRLL
PVLCQAHGLTPEQVVAIASNIGGKQALETVQRLLPVLCQAHGLTPDQVVAIASNNGGKQ
ALETVQRLLPVLCQAHGLTPEQVVAIASHDGGKQALETVQRLLPVLCQAHGLTPAQVVA
IASHDGGKQALETVQRLLPVLCQAHGLTPAQVVAIASHDGGKQALETVQRLLPVLCQAH
GLTPAQVVAIASNIGGKQALETVQRLLPVLCQAHGLTPDQVVAIASHDGGKQALETVQR
LLPVLCQAHGLTPAQVVAIASNGGGKQALETVQRLLPVLCQAHGLTPEQVVAIASNNGG
KQALETVQRLLPVLCQAHGLTPEQVVAIASNGGGKQALETVQRLLPVLCQAHGLTPEQV
VAIASNIGGKQALETVQRLLPVLCQAHGLTPDQVVAIASNIGGKQALETVQRLLPVLCQA
HGLTPDQVVAIASNIGGKQALETVQRLLPVLCQAHGLTPDQVVAIASNNGGKQALETVQ
RLLPVLCQAHGLTPEQVVAIASHDGGKQALETVQRLLPVLCQAHGLTPAQVVAIASNGG
GRPALESIVAQLSRPDPALA

Human m.G8313 Right TALE:

LTPEQVVNAIASGGGKQALETVQRLLPVLCQAHGLTPEQVVAIASNIGGKQALETVQRLL
PVLCQAHGLTPDQVVAIASNIGGKQALETVQRLLPVLCQAHGLTPDQVVAIASNGGGKQ
ALETVQRLLPVLCQAHGLTPEQVVAIASHDGGKQALETVQRLLPVLCQAHGLTPAQVVA
IASNGGGKQALETVQRLLPVLCQAHGLTPEQVVAIASNGGGKQALETVQRLLPVLCQAH
GLTPEQVVAIASNGGGKQALETVQRLLPVLCQAHGLTPEQVVAIASNIGGKQALETVQRL
LPVLCQAHGLTPDQVVAIASNIGGKQALETVQRLLPVLCQAHGLTPDQVVAIASHDGGK
QALETVQRLLPVLCQAHGLTPAQVVAIASNGGGKQALETVQRLLPVLCQAHGLTPEQVV
AIASNGGGKQALETVQRLLPVLCQAHGLTPEQVVAIASNIGGKQALETVQRLLPVLCQA
HGLTPDQVVAIASNIGGKQALETVQRLLPVLCQAHGLTPDQVVAIASNIGGRPALESIVAQ
LSRPDPALA

Human n.JAK2 Left TALE:

LTPEQVVAIASHDGGKQALETVQRLLPVLCQAHGLTPAQVVAIASNGGGKQALETVQRL
LPVLCQAHGLTPEQVVAIASNNGGKQALETVQRLLPVLCQAHGLTPEQVVAIASNIGGK
QALETVQRLLPVLCQAHGLTPDQVVAIASNIGGKQALETVQRLLPVLCQAHGLTPDQVV
AIASNIGGKQALETVQRLLPVLCQAHGLTPDQVVAIASNIGGKQALETVQRLLPVLCQAH
GLTPDQVVAIASNIGGKQALETVQRLLPVLCQAHGLTPDQVVAIASNNGGKQALETVQR
LLPVLCQAHGLTPEQVVAIASNIGGKQALETVQRLLPVLCQAHGLTPDQVVAIASHDGG
KQALETVQRLLPVLCQAHGLTPAQVVAIASNGGGKQALETVQRLLPVLCQAHGLTPEQV
VAIASHDGGKQALETVQRLLPVLCQAHGLTPAQVVAIASNGGGKQALETVQRLLPVLCQ
AHGLTPEQVVAIASNNGGKQALETVQRLLPVLCQAHGLTPEQVVAIASHDGGKQALETV
QRLLPVLCQAHGLTPAQVVAIASNIGGRPALESIVAQLSRPDPALA

Human n.JAK2 Right TALE:

LTPEQVVAIASHDGGKQALETVQRLLPVLCQAHGLTPAQVVAIASHDGGKQALETVQRL
LPVLCQAHGLTPAQVVAIASNIGGKQALETVQRLLPVLCQAHGLTPDQVVAIASNGGGK
QALETVQRLLPVLCQAHGLTPEQVVAIASNGGGKQALETVQRLLPVLCQAHGLTPEQVV
AIASNGGGKQALETVQRLLPVLCQAHGLTPEQVVAIASHDGGKQALETVQRLLPVLCQA
HGLTPAQVVAIASNGGGKQALETVQRLLPVLCQAHGLTPEQVVAIASNNGGKQALETVQ
RLLPVLCQAHGLTPEQVVAIASNGGGKQALETVQRLLPVLCQAHGLTPEQVVAIASHDG
GKQALETVQRLLPVLCQAHGLTPAQVVAIASNIGGKQALETVQRLLPVLCQAHGLTPDQ
VVAIASNGGGKQALETVQRLLPVLCQAHGLTPEQVVAIASHDGGKQALETVQRLLPVLC
QAHGLTPAQVVAIASNNGGKQALETVQRLLPVLCQAHGLTPEQVVAIASNGGGKQALET
VQRLLPVLCQAHGLTPEQVVAIASNIGGRPALESIVAQLSRPDPAL

Human m.A8344G Left TALE #6:

LTPEQVVAIASNGGGKQALETVQRLLPVLCQAHGLTPEQVVAIASNIGGKQALETVQRLL
PVLCQAHGLTPDQVVAIASNIGGKQALETVQRLLPVLCQAHGLTPDQVVAIASNNGGKQ
ALETVQRLLPVLCQAHGLTPEQVVAIASNGGGKQALETVQRLLPVLCQAHGLTPEQVVA
IASNGGGKQALETVQRLLPVLCQAHGLTPEQVVAIASNIGGKQALETVQRLLPVLCQAH
GLTPDQVVAIASNIGGKQALETVQRLLPVLCQAHGLTPDQVVAIASNIGGKQALETVQRL
LPVLCQAHGLTPDQVVAIASNNGGKQALETVQRLLPVLCQAHGLTPEQVVAIASNIGGK
QALETVQRLLPVLCQAHGLTPDQVVAIASNGGGKQALETVQRLLPVLCQAHGLTPEQVV
AIASNGGGKQALETVQRLLPVLCQAHGLTPEQVVAIASNIGGKQALETVQRLLPVLCQA
HGLTPDQVVAIASNIGGKQALETVQRLLPVLCQAHGLTPDQVVAIASNNGGRPALESIVA
QLSRPDPAL

Human m.A8344G Right TALE #6:

LTPEQVVAIASNNGGKQALETVQRLLPVLCQAHGLTPEQVVAIASNNGGKQALETVQRL
LPVLCQAHGLTPEQVVAIASNNGGKQALETVQRLLPVLCQAHGLTPEQVVAIASNNGGK
QALETVQRLLPVLCQAHGLTPEQVVAIASHDGGKQALETVQRLLPVLCQAHGLTPAQVV
AIASNIGGKQALETVQRLLPVLCQAHGLTPDQVVAIASNGGGKQALETVQRLLPVLCQA
HGLTPEQVVAIASNGGGKQALETVQRLLPVLCQAHGLTPEQVVAIASNGGGKQALETVQ
RLLPVLCQAHGLTPEQVVAIASHDGGKQALETVQRLLPVLCQAHGLTPAQVVAIASNIGG
KQALETVQRLLPVLCQAHGLTPDQVVAIASHDGGKQALETVQRLLPVLCQAHGLTPAQV
VAIASNGGGKQALETVQRLLPVLCQAHGLTPEQVVAIASNNGGKQALETVQRLLPVLCQ
AHGLTPEQVVAIASNGGGKQALETVQRLLPVLCQAHGLTPEQVVAIASNIGGKQALETV
QRLLPVLCQAHGLTPDQVVAIASNIGGKQALETVQRLLPVLCQAHGLTPDQVVAIASNIG
GKQALETVQRLLPVLCQAHGLTPDQVVAIASNNGGKQALETVQRLLPVLCQAHGLTPEQ
VVAIASNIGGRPALESIVAQLSRPDPAL

DddA-DddI$_A$:

MYADDFDGEIEIDEVDSLVEFLSRRPAFDANNFVLTFEESGFPQLNIFAKNDIAVVYYMDI
GENFVSKGNSASGGTEKFYENKLGGEVDLSKDCVVSKEQMIEAAKQFFATKQRPEQLT
WSEL

FZY2-DddI$_A$:

MLVEHFMGQKECDSLEELREVLSERTEKGVNEFIISTHEQFPYMIMSVKEKYACLSYFRE
EDDPGYSSVNANPVLDADGISIFYTNTDSEEIEVANYSIVKIEDAVSAVEEFFETLQLPKCI
EWEEL

WC03-DddI$_A$:

MKIKHFGGIEYANNISEFSEILQKKYGDGVNEFWITNNTQENPCLVVLVNKEMANLTYFP
DEESLGFQSVGCQTNQKNDYCIFYTNTPEEEIEIRRDSIISVDKAFEAAKKFFYKREMPDN
IEWTEN

L9D3-DddI$_A$:

MIEALKMQYFGGACEMHARQELEAALAQWSGVANDYWLSHGNAKFPALAVMANGPL
ATAHYFPDARHPGFISIGNVNGLDPEGTSTFFLTDTETTDVANYRVIPIEVAWEAAREFAEC
SRRPNCIEWIEL

XG57-DddI$_A$:

MMEVMKMQYFGGACKMRTREEVDAALARWSGAANDYWLSHGNAKFPALAVMVNGQ
LATAHYFPDEHHPGFISLGNVNGLDPDGTSTFFLTNTETTDVANERVISIEVAWEAAREFA
QNWSLPRCIKWFEL

HR14-DddI$_A$:

MAKRKCGALVVMVQLVLTSNDGRTEQETAAPSKQDVESRIAGLDGIERDLVILYRNDSH
FAVGGSASGRLVASCTFDNQELGQLTSGGDPDAEITVMAGRQAGDYPANQVVGLAEVLT
AVDAFVEDGSLAVKVSWKRS
