# Supplementary material for: Developing mitochondrial base editors with diverse context compatibility and high fidelity via saturated spacer library
Source: Nat Commun. 2023 Oct 19;14:6625. doi: 10.1038/s41467-023-42359-3 (PMC10587121; doi:10.1038/s41467-023-42359-3)
Supplement: Supplementary file 2 — Reporting Summary [file 41467_2023_42359_MOESM2_ESM.pdf]

nature portfolio

Corresponding author(s): Bin Shen, Jun Zhang, Xin Lou

Last updated by author(s): Sep 24, 2023

Reporting Summary

Nature Portfolio wishes to improve the reproducibility of the work that we publish. This form provides structure for consistency and transparency in reporting. For further information on Nature Portfolio policies, see our Editorial Policies and the Editorial Policy Checklist.

Statistics

For all statistical analyses, confirm that the following items are present in the figure legend, table legend, main text, or Methods section.

n/a	Confirmed	
☐	☒	The exact sample size (*n*) for each experimental group/condition, given as a discrete number and unit of measurement
☐	☒	A statement on whether measurements were taken from distinct samples or whether the same sample was measured repeatedly
☐	☒	The statistical test(s) used AND whether they are one- or two-sided<br>*Only common tests should be described solely by name; describe more complex techniques in the Methods section.*
☒	☐	A description of all covariates tested
☒	☐	A description of any assumptions or corrections, such as tests of normality and adjustment for multiple comparisons
☐	☒	A full description of the statistical parameters including central tendency (e.g. means) or other basic estimates (e.g. regression coefficient) AND variation (e.g. standard deviation) or associated estimates of uncertainty (e.g. confidence intervals)
☐	☒	For null hypothesis testing, the test statistic (e.g. *F*, *t*, *r*) with confidence intervals, effect sizes, degrees of freedom and *P* value noted<br>*Give P values as exact values whenever suitable.*
☒	☐	For Bayesian analysis, information on the choice of priors and Markov chain Monte Carlo settings
☒	☐	For hierarchical and complex designs, identification of the appropriate level for tests and full reporting of outcomes
☒	☐	Estimates of effect sizes (e.g. Cohen's *d*, Pearson's *r*), indicating how they were calculated

Our web collection on statistics for biologists contains articles on many of the points above.

Software and code

Policy information about availability of computer code

Data collection	High-throughput sequencing (HTS) data were collected by the Illumina NovaSeq.
Data analysis	HTS data were analyzed with bowtie2 (v2.3.4.1), samtools (v1.15) and homemade python (v3.7.5) or R (v3.4.4) scripts. Mean and standard deviations (SD) were calculated using GraphPad Prism 9. All codes are available upon request.

For manuscripts utilizing custom algorithms or software that are central to the research but not yet described in published literature, software must be made available to editors and reviewers. We strongly encourage code deposition in a community repository (e.g. GitHub). See the Nature Portfolio guidelines for submitting code & software for further information.

Data

Policy information about availability of data

All manuscripts must include a data availability statement. This statement should provide the following information, where applicable:

- Accession codes, unique identifiers, or web links for publicly available datasets
- A description of any restrictions on data availability
- For clinical datasets or third party data, please ensure that the statement adheres to our policy

High-throughput sequencing (HTS) data generated in this study have been deposited in the Sequence Read Archive (SRA) database under accession code PRJNA938742.

Research involving human participants, their data, or biological material

Policy information about studies with human participants or human data. See also policy information about sex, gender (identity/presentation), and sexual orientation and race, ethnicity and racism.

Reporting on sex and gender	n/a
Reporting on race, ethnicity, or other socially relevant groupings	n/a
Population characteristics	n/a
Recruitment	n/a
Ethics oversight	Human amniotic fluid cells (AFCs) were collected from leftover samples during prenatal diagnosis. Written informed consent from the pregnant woman was obtained for using the AFCs for research. This study was approved by the ethics review board of Women's Hospital Affiliated to Nanjing Medical University (2016KY-87).

Note that full information on the approval of the study protocol must also be provided in the manuscript.

Field-specific reporting

Please select the one below that is the best fit for your research. If you are not sure, read the appropriate sections before making your selection.

☒ Life sciences ☐ Behavioural & social sciences ☐ Ecological, evolutionary & environmental sciences

For a reference copy of the document with all sections, see nature.com/documents/nr-reporting-summary-flat.pdf

Life sciences study design

All studies must disclose on these points even when the disclosure is negative.

Sample size	No sample-size calculation was performed. All cell samples were evaluated in at least two independent replicate experiments to ensure the reproducibility and the sample size numbers were listed in the corresponding figure legends. Sample sizes were chosen in accordance with standards in the field that have been sufficient to develop and validate base editing tools.
Data exclusions	No data were excluded.
Replication	All base editing experiments were repeated with at least two biological repeats to confirm reproducibility. All attempts at reproducibility were successful, and standard deviations were indicated in the figure legends.
Randomization	Cells were randomly assigned into culture plate for transfection.
Blinding	Not applicable. All experiments have been processed and analyzed in an unbiased way. Analysis did not require blinding.

Reporting for specific materials, systems and methods

We require information from authors about some types of materials, experimental systems and methods used in many studies. Here, indicate whether each material, system or method listed is relevant to your study. If you are not sure if a list item applies to your research, read the appropriate section before selecting a response.

Materials & experimental systems

n/a	Involved in the study	
☒	☐	Antibodies
☐	☒	Eukaryotic cell lines
☒	☐	Palaeontology and archaeology
☒	☐	Animals and other organisms
☒	☐	Clinical data
☒	☐	Dual use research of concern
☒	☐	Plants

Methods

n/a	Involved in the study	
☒	☐	ChIP-seq
☐	☒	Flow cytometry
☒	☐	MRI-based neuroimaging

Eukaryotic cell lines

Policy information about cell lines and Sex and Gender in Research

Cell line source(s)	HEK293FT cells were obtained from Thermofisher (R70007). Human amniotic fluid cells (AFCs) were collected from leftover samples during prenatal diagnosis and immortalized using SV40 large T antigen lentivirus.
Authentication	No specific authentication was performed.
Mycoplasma contamination	All cell lines were detected without mycoplasma contamination by PCR test.
Commonly misidentified lines (See ICLAC register)	No commonly misidentified cell lines were used.

Flow Cytometry

Plots

Confirm that:

- [x] The axis labels state the marker and fluorochrome used (e.g. CD4-FITC).
- [x] The axis scales are clearly visible. Include numbers along axes only for bottom left plot of group (a 'group' is an analysis of identical markers).
- [x] All plots are contour plots with outliers or pseudocolor plots.
- [x] A numerical value for number of cells or percentage (with statistics) is provided.

Methodology

Sample preparation	The immortalized human amniotic fluid cells were trypsinized, resuspended in culture medium and sorted via flow cytometry.
Instrument	BD FACSAria™ Fusion SORP sorter
Software	FACS DIVA 8.0
Cell population abundance	EGFP and mCherry double positive rates = double positive cell number / total cell number.
Gating strategy	The cells were first gated based on FSC/SSC and FSC-A/FSC-H to select for live single cells. Untreated cells were employed as negative control for gating EGFP and mCherry signal.

- [x] Tick this box to confirm that a figure exemplifying the gating strategy is provided in the Supplementary Information.
