# Supplementary material for: Developing mitochondrial base editors with diverse context compatibility and high fidelity via saturated spacer library
Source: Nat Commun. 2023 Oct 19;14:6625. doi: 10.1038/s41467-023-42359-3 (PMC10587121; doi:10.1038/s41467-023-42359-3)
Supplement: Supplementary file 3 — Description of Additional Supplementary Files [file 41467_2023_42359_MOESM3_ESM.pdf]

Title: Supplementary Data 1

Description: This file provides the information of all DddA homologs, including their Accession, Rechristened Name, Protein Name, Tax Name and Amino acid Sequences in sheet 1. In sheet 2, RMSD (root mean square deviation) values between DddA and its homologs are provided.

Title: Supplementary Data 2

Description: This file provides the information of all $DddI_A$ homologs, including their Accession, Rechristened Name, Tax ID, Tax Name and Amino acid Sequences.
